# Molecular Insights into miRNA-Driven Resistance to 5-Fluorouracil and Oxaliplatin Chemotherapy: miR-23b Modulates the Epithelial–Mesenchymal Transition of Colorectal Cancer Cells

**DOI:** 10.3390/jcm8122115

**Published:** 2019-12-02

**Authors:** Stasė Gasiulė, Nadezda Dreize, Algirdas Kaupinis, Raimundas Ražanskas, Laurynas Čiupas, Vaidotas Stankevičius, Žana Kapustina, Arvydas Laurinavičius, Mindaugas Valius, Giedrius Vilkaitis

**Affiliations:** 1 Institute of Biotechnology, Life Sciences Center, Vilnius University, Vilnius LT-10257, Lithuania; stase.gasiule@bti.vu.lt (S.G.); raimundas.razanskas@bti.vu.lt (R.R.); ciupas.laurynas@gmail.com (L.Č.); vaidotas.stankevicius@gmc.vu.lt (V.S.); 2 Institute of Biochemistry, Life Sciences Center, Vilnius University, Vilnius LT-10257, Lithuania; nadezda.dreize@gmc.vu.lt (N.D.); algirdas.kaupinis@gf.vu.lt (A.K.); 3 Thermo Fisher Scientific Baltics, Vilnius LT-02241, Lithuania; zana.kapustina@thermofisher.com; 4 National Center of Pathology, Affiliate of Vilnius University Hospital Santaros Klinikos, Vilnius LT-08406, Lithuania; Arvydas.Laurinavicius@vpc.lt; 5 Faculty of Medicine, Vilnius University, Vilnius LT-03101, Lithuania

**Keywords:** oxaliplatin, 5-fluorouracil, chemoresistance, microRNA, miRNome, proteome, epithelial–mesenchymal transition, drug resistance

## Abstract

Although treatment of colorectal cancer with 5-florouracil and oxaliplatin is widely used, it is frequently followed by a relapse. Therefore, there is an urgent need for profound understanding of chemotherapy resistance mechanisms as well as the profiling of predictive markers for individualized treatment. In this study, we identified the changes in 14 miRNAs in 5-fluouracil and 40 miRNAs in oxaliplatin-resistant cell lines by miRNA sequencing. The decrease in miR-224-5p expression in the 5-fluorouracil-resistant cells correlated with drug insensitivity due to its overexpression-induced drug-dependent apoptosis. On the other hand, the miR-23b/27b/24-1 cluster was overexpressed in oxaliplatin-resistant cells. The knockout of miR-23b led to the partial restoration of oxaliplatin susceptibility, showing the essential role of miR-23b in the development of drug resistance by this cluster. Proteomic analysis identified target genes of miR-23b and showed that endothelial–mesenchymal transition (EMT) was implicated in oxaliplatin insensibility. Data revealed that EMT markers, such as vimentin and SNAI2, were expressed moderately higher in the oxaliplatin-resistant cells and their expression increased further in the less drug-resistant cells, which had miR-23b knockout. This establishes that the balance of EMT contributes to the drug resistance, showing the importance of the miR-23b-mediated fine-tuning of EMT in oxaliplatin-resistant cancer cells.

## 1. Introduction

The majority of chemotherapeutic drugs do not kill a whole pool of heterogenic cancerous cells. Some of them survive and acquire resistance to therapy treatment, thus leading to the recurrence of malignance. Unfortunately, data obtained from cancer tissues before treatment do not always lead to the prediction of drug tolerance mechanisms, since the data from minor drug-resistant population are masked by background species from a pool of non-resistant cells [1]. One way to overcome this limitation and to discover relevant predictive markers is to generate and analyze sublines of tumor cultures in vitro, which are more homogeneous with respect to genetic population and enable the determination of drug-resistance mechanisms under the more controlled in vitro conditions.

The combinations of 5-fluorouracil (5-FU) and oxaliplatin (Oxa) drugs often supplemented with synergizing medications, such as leucovorin in the case of FOLFOX regimen, or folinic acid in FLOX regimen, are adjuvant chemotherapy schemes commonly used for treatment of patients with surgically resected colon cancer (CRC) [2,3]. The combination of Oxa and capecitabine represents an alternative CAPOX regimen, which could be used for colon cancer treatment [4]. Many different biological processes as well as their combinations can be involved in the acquisition of 5-FU- or Oxa-resistance. These include drug metabolism, restriction of drug entrance, increase in drug efflux, DNA damage repair, changes in autophagy flux, cancer stem cells proliferation, epigenetic effects, and endothelial–mesenchymal transition (EMT) [5,6,7]. Most of these mechanisms can be triggered or at least influenced by small non-coding RNAs, such as microRNAs (miRNAs), and long noncoding RNAs (lncRNAs) via the involvement of massive alterations in gene expression [7].

The 5-FU anticancer action mainly depends on the thymidylate synthase (TS) and dihydropyrimidine dehydrogenase (DPYD) pathways. Therefore, miR-197, -203, and -218 that effect TS expression and miR-27a, -27b, -200c, and -494 that regulate DPYD activity stringently control cytotoxicity of the drug [5,8]. However, there is evidence that let-7, miR-20b, -135b, -182, -200c, -204, -224, and -587 miRNAs impair 5-FU sensitivity by regulating proteins related to the PI3K/AKT signaling pathway. On the other hand, miR-125b, -149, -320, -181a-5p and lncRNA CRNDE change cancer cell resistance to 5-FU by controlling the Wnt/β-catenin signaling pathway [5].

Much less is known about miRNA-driven molecular mechanisms responsible for the development of resistance to the Oxa treatment. Perturbations in miR-520g expression affects the efficiency of the treatment via the cyclin-dependent kinase inhibitor p21-dependent pathway [9]. On the other hand, miR-20a regulates Bcl-2-dependent apoptosis via BNIP2 [10]. Changes in miR-153 expression mediate Oxa resistance by inhibiting the transcription factor Forkhead box O3a (FOXO3a) [11], and miR-203 modulates DNA damage response via Ataxia Telangiectasia Mutated (ATM) regulation [12]. Notably, some miRNA-mediated effects require changes in the genetic background, such as KRAS or TP53 mutations [9,13], once again suggesting multi-variant mechanisms of cancer cell drug-resistance acquisition.

EMT in cancerous cells emerges among the major hallmarks for cancerogenesis and drug-resistance. Cells undergoing EMT show a feature similar to cancer stem cells, such as an increase in the drug efflux and anti-apoptotic properties [14]. On the other hand, the plasticity of EMT as recently recognized mesenchymal–endothelial transition (MET) reversal of the EMT phenotype associated with the lack of stringent and widely recognized markers of those cell states creates a complicated picture of EMT–MET phenomena. The picture becomes even more complex after acknowledgment that an intermediate state of EMT contributes mostly to the cancer cell dissemination and adaptation to the new homing (metastatic) conditions [15]. Moreover, it has been shown that a few gene expressions related to this status, including *SNAI2*, play a decisive role in cancer cell drug resistance rather than the EMT phenotype per se [16,17,18].

**Figure 1:**
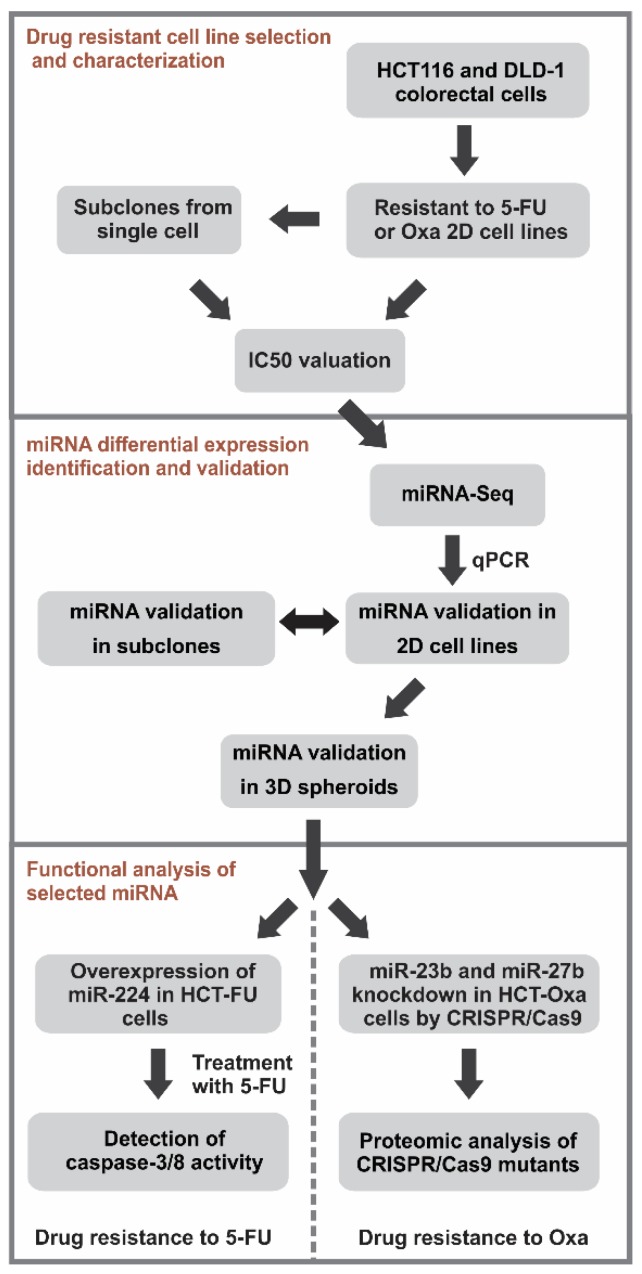
The scheme of experimental design. At first, four drug-resistant cell sublines were generated from HCT116 and DLD-1 cells by continuous and pulse exposure to 5-FU or Oxa. In the next stage, differential miRNA expression profiles were determined in two drug-resistant sublines, HCT-Oxa-c and HCT-FU-c. Selected miRNAs were validated by qPCR in 2D cells and 3D spheroids. Finally, we analyzed the functional effect of the overexpression of miR-224-5p, which level was decreased in 5-FU-resistant lines. The knockout of miR-23b and miR-27b allowed us to define the roles of these miRNAs in Oxa-resistant lines. The molecular mechanism of resistance to Oxa was then determined by proteomic analysis of HCT116, HCT-Oxa-c and 23b-/- mutants.

In this study, we have applied four in vitro generated 5-FU or Oxa-resistant colorectal cancer cell lines to decipher the networks of differentially expressed miRNAs by miRNA-Seq, which potentially regulates resistance to the FOLFOX regiment (Figure 1). Expression changes in these miRNAs were confirmed in the 2D as well as in the 3D cultures. The role of the miR-224/452 cluster was identified as a guardian of 5-FU-induced apoptosis in the drug-resistant cells. Alternatively, the miR-23b/27b/24-1 cluster was implicated in resistance to Oxa by partially changing the epithelial–mesenchymal transition of Oxa-resistant CRC cells.

## 2. Materials and Methods

### 2.1. Cell Culture and Establishment of Resistant Cell Lines

Human colorectal carcinoma HCT116 and human colorectal adenocarcinoma DLD-1 cells were maintained in Dulbecco’s modified Eagle’s medium (DMEM) (ThermoFisher Scientific) supplemented with 10% fetal bovine serum (FBS) (ThermoFisher Scientific) and penicillin-streptomycin antibiotics (ThermoFisher Scientific).

Resistant HCT116 cells were selected by the two different methods—that is, by using continuous or the pulsed exposures to oxaliplatin (Oxa) (Accord) or 5-fluorouracil (5-FU) (Teva). Continuous exposure was achieved by continuously culturing HCT116 cells in a medium containing increasing concentrations of 5-FU) or (Oxa), as described in Dabkeviciene et al. [19] and Kukcinaviciute et al. [20] and sublines were labeled HCT-Oxa-c and HCT-FU-c, respectively. Considering the clinical application of the FOLFOX protocol, parental cells were also subjected to the pulsed exposures. According to the FOLFOX protocol, cells were treated for 2 h with oxaliplatin or for 48 h with 5-fluorouracil followed by the cell recovery stage. Drug treatment was continued for approximately 9 months until the cells acquired stable drug resistance. DLD-1-resistant cell lines were selected only by pulsed exposure resembling FOLFOX treatment. HCT-Oxa-p and DLD-1-p sublines were generated by using pulsed selection.

### 2.2. Cell Viability Evaluation in the 2D Cell Culture

Resistance of the parental and resistant cell lines to 5-fluorouracil or oxaliplatin was evaluated using MTT (Roth) assay. The day before treatment, cells were seeded in 96-well flat-bottomed plates (3000 cells/well). Cells were treated for 48 h with increasing drug concentrations. After treatment, fresh growth media was applied and the MTT test was performed 96 h after beginning the treatment. A medium with 0.5 mg/mL MTT was added to each well and incubated for 45 min at 37 °C. The formazan product was dissolved by adding 100 μL dimethylsulfoxide (Roth) to each well, and the plates were red at 570 nm with Varioskan Flash Multimode Reader (ThermoFisher Scientific). Results were normalized according to untreated control and changes between cell lines were evaluated. All measurements were performed in quadruplicate and each experiment was repeated at least three times.

### 2.3. RNA Isolation

Total RNA from cell lines and spheroids was isolated using an RNAzol^®^ RT (Molecular Research Center) reagent according to the manufacturer’s instructions. 32 spheroids were used to isolate RNA. RNA purity and concentration were assessed using NanoDrop (ThermoFisher Scientific). The integrity was assessed using an Agilent 2100 Bioanalyzer using Agilent RNA 6000 Nano Kit (Agilent) or agarose (1%) gel electrophoresis. To avoid DNA contamination before reverse transcription reaction, RNA samples were treated with DNase I (ThermoFisher Scientific) according to the manufacturer’s instructions.

### 2.4. cDNA Library Preparation for Illumina Sequencing

Complementary DNA (cDNA) libraries for sequencing were prepared using NEXTflex Small RNA-Seq v3 kit (PerkinElmer) according to the manufacturer’s instructions. Ligation reactions of 3′ and 5′ adapters were performed using 1.2 µg total RNA. The cDNA was amplified 16 PCR cycles. PCR products were analyzed with Bioanalyzer (Agilent technologies) using DNA High sensitivity chip (Agilent technologies) and were quantified by KAPA Library Quantification Kit (Rosche). Libraries pooled for multiplexed sequencing (5–6 libraries per run), hybridized to Illumina MiSeq flow cell (Illumina) and subsequently sequenced using Illumina MiSeq instrument and MiSeq Reagent Kit v2 chemistry (Illumina) for 50 sequencing cycles.

### 2.5. Quantitative Reverse Transcription PCR

For miRNA qPCR and for *AP-O*, *GABRE*, *Vimentin*, *SNAI2* gene expression 1 µg total RNA was used for cDNA synthesis. miRNA cDNA reaction conditions were described previously [21]. cDNA from mRNA were prepared according to the manufacturer’s instructions. Real-time PCR was performed with SYBR Green PCR master mix (ThermoFisher Scientific) according to the manufacturer’s instructions. Relative quantification of changes in the miRNA and gene expression levels was performed using the comparative C_t_ (threshold cycle) method with normalization to the expression of endogenous control *RNU48*, *GUS* or *GAPDH*. Primers (Metabion) are provided in Supplementary Table S15. Quantitative PCR analysis was carried out on Rotor-Gene 6000 (Corbett Life Science) equipment.

### 2.6. Analysis of miR-224 Overexpression Effect on 5-FU Resistance

HCT-FU-c cell line cells (2 × 10^5^ cells/well) were seeded on the glass surface of a microscopy holder 24 h prior to transfection. Cells were transfected with mock or miR-224 harboring overexpression vectors using Lipofectamine LTX Transfection reagent (Thermo Fisher Scientific) according to the manufacturer’s instructions. Growth media was changed after 24 h, then 2 µM Cell Event™ Caspase-3/7 Green Detection Reagent (Thermo Fisher Scientific) was added and cells were incubated in 37 ℃ in the dark for 30 min. Cells were treated with 25 µM 5-FU (the concentration is comparable to IC20 of HCT-FU-c cell line) and fluorescence signals of 5–9 different locations were registered in 15 min intervals for 48 h. The fluorescence of cells was registered with laser scanning microscope ZEISS LSM 710 using 20 × 1.25 N/A lenses at 37 ℃, 5% CO2 and 95% humidity atmosphere.

Two fluorescence signals were registered: green (Cell Event dye—cells with activated caspase 3/7) and red (mCherry dye—a marker for miRNA-transfected cells). Cell Event dye was excited using 488 nm and the emitted signal was registered up to 548 nm. mCherry fluorescence was excited using 560 nm and registered at 653–700 nm.

Data were analyzed using ZEN (Zeiss) and ImageJ programs. All cells were counted and some of the transfected cells population (red signal) as well as activated caspase 3/7 (green signal) were evaluated by merging two channels and recording the yellow signal (transfected cells with activated caspase 3 and 7). Time points in which caspase 3 and 7 were activated in transfected cells were determined for every individual cell (when red cell becomes yellow or green). Acquired results at different time points were summed up and caspase 3/7 activation kinetics were determined during 48 h period.

### 2.7. Generation of the Cell Lines with the Down-Regulated Expression of miR-23b or miR-27b Using CRISPR/Cas9

The sgRNA-23b and sgRNA-27b were designed using the Benchling program (Supplementary Figure S7) and inserted into pSpCas9(BB)-2A-Puro (Addgene 62988) as described previously [22]. HCT-Oxa-c cells (15 × 10^4^ cells/well) were seeded in 24-well plates 24 h before transfection and constructs were transfected into HCT-Oxa-c cells using Lipofectamine LTX (Thermo Fisher Scientific) according to the manufacturer’s instructions. After 48 h of transfection, cells were incubated for 48–72 h with 2 µg/mL puromycin (Sigma). Then puromycin-resistant cells were isolated through serial dilutions, seeding one cell per well of 96-well plates. Cells were grown in an incubator for 1–2 weeks, when cultivated into 24-well plates. Some of the cells were collected into tubes and indels were detected as described previously using polyacrylamide gel electrophoresis [23]. PCR was carried out with the Phire Tissue Direct PCR master Mix (Thermo Fisher Scientific). The list of primers and single-guide RNA (sgRNA) sequences (Metabion) are provided in Supplementary Table S15.

### 2.8. Proteomic Analysis

For proteomic analysis, HCT116, HCT-Oxa-c and miR-23b knockdown cell lines were used. Cells were lysed with 0.5 mL of urea/thiourealysis buffer (7M urea (GE Healthcare), 2M thiourea (GE Healthcare), 40 mM DTT (Sigma)). The lysates were sonicated for 1 min at an amplitude of 20% and 0.4 s pulsation on/off cycles (Sonopuls HD 2070, Bandelin, Germany). Homogenized lysates were centrifuged at 20,000 g for 15 min at 4 °C, and the supernatants were collected. The lysates then were stored at −86 °C.

Trypsin (ThermoFisher Scientific) digestion was performed according to a modified filter-aided sample preparation (FASP) protocol as described by Wisniewski et al. [24]. LCMS data were collected as described previously [25]. Briefly, liquid chromatographic analysis was performed in a Waters Acquity Ultra Performance LC system (Waters Corporation, Wilmslow, UK). Peptide separation was performed on an ACQUITY UPLC HSS T3 250 mm analytical column (Merk Milipore). Data were acquired using Synapt G2 mass spectrometer and Masslynx 4.1 software (Waters Corporation) in positive ion mode using data independent (DIA) acquisition (UDMSE acquisition mode) (22). Raw data were lock mass-corrected using the doubly charged ion of Glu1-fibrinopeptide B (m/z 785.8426; (M+2H) 2+). The proteomic data are available via MassIVE database with the identifier MSV000083286.

Raw data files were processed and searched using ProteinLynx Global SERVER version 3.0.1 (Waters Corporation, UK). Data were analyzed using trypsin as the cleavage protease; one missed cleavage was allowed and fixed modification was set to carbamidomethylation of cysteines, and variable modification was set to oxidation of methionine. Minimum identification criteria included 1 fragment ion per peptide, 3 fragment ions per protein and a minimum of 2 peptides per protein. The following parameters were used to generate peak lists: (i) minimum intensity for precursors was set to 135 counts; (ii) minimum intensity for fragment ions was set to 25 counts; (iii) intensity was set to 750 counts. UniprotKB/SwissProt human database (20 Sep.2018) was used for protein identification.

Label-free quantification (TOP3) was performed using ISOQuant software [26]. A differential expression analysis was performed using the linear models approach of the piano package [27]. Proteins were considered differentially expressed if the protein level was statistically significantly (*p* ≤ 0.01) increased or decreased.

### 2.9. Confocal Microscopy

Immunofluorescence experiments were performed on cells grown in 24-well plates on glass coverslips. Cells were fixed with 4% paraformaldehyde (Roth) in PBS (pH 7.4), permeabilized with 0.2% Triton X-100 (Roth) and stained with anti-vimentin clone RV202 (BD Pharmingen), followed by secondary Alexa Fluor 488-conjugated anti-mouse IgG (ThermoFisher Scientific). Cell nuclei were stained with 300 nM DAPI dye (ThermoFisher Scientific). Specimens were analyzed with a laser scanning spectral confocal microscope (Eclipse TE2000-S, C1 plus, Nikon) with Apo TIRF 60 × N/A 1.4 objective (Nikon).

### 2.10. Statistical and Bioinformatics Analysis

Statistical analysis was performed using SigmaPlot software v. 12. The unpaired Student’s t test and Mann–Whitney rank sum test were used to compare the differences in distribution between biological replicates. A value of *p* < 0.05 was considered statistically significant.

miRNA-Seq data are available at the GEO database using accession number GSE119481. Detailed differential miRNA-Seq data on each sample are presented in Supplementary File 1. Proteomic datasets of each sample are shown in Supplementary Files 2 and 3.

Cell viability evaluation in the 3D cell culture, miRNA-Seq differential and functional analysis, miRNA target analysis, computational functional analysis of proteomic data, and wound healing assay are described in detail in the Supplementary Methods section of the Supplementary data.

## 3. Results

### 3.1. In vitro Generation and Characterization of 5-Fluorouracil- or Oxaliplatin-Resistant Cell Sublines

Primary analysis showed that 5-fluorouracil (5-FU) and oxaliplatin (Oxa) drugs at the low-micromole concentrations effectively reduced the viability of the parental colorectal carcinoma epithelial cells lines: 10.5 versus 11.2 µM cytotoxicity half maximal inhibitory concentration (IC50) values for 5-FU and 4.1 versus 31.7 µM for oxaliplatin were determined for HCT116 and DLD-1, respectively. Drug-resistant cells have been selected by: (i) continuous treatment of cell culture with the increasing concentration of the drug of interest with no recovery phase; (ii) pulse treatment with increasing concentration of drug with subsequent drug-free cultivation to allow cells to recover. We have anticipated that using different protocols of selection enable us to approximate variations of in vitro procedures and, therefore, bring selection of drug resistance closer to the in vivo situation.

Four sublines of resistant cells were established by continuous (c) and pulse (p) exposure to the 5-fluorouracil or oxaliplatin compound. Two of them, HCT-FU-c and DLD-FU-p, gained strongly pronounced resistance to 5-fluouracil treatment, whereas HCT-Oxa-c and HCT-Oxa-p gained strongly pronounced resistance to oxaliplatin (Supplementary Table S1; Supplementary Figures S1 and S2).

### 3.2. High-Throughput Sequencing Analysis of miRNA Expression Profiles of the Drug-Resistant Sublines

To investigate the impact of miRNAs on the emerged insensitivity to the 5-FU or Oxa treatment, we performed a sequencing of small RNA libraries from the HCT-Oxa-c and HCT-FU-c cell lines and determined miRNA changes compared to the parental HCT116 line (Supplementary Table S2). As shown in Supplementary Table S3, 40 and 14 of miRNAs were differentially expressed with high confidence relative to the parental cell line, thereby potentially contributing to drug adaptation in HCT-Oxa-c and HCT-FU-c sublines, respectively. Only two of them, miR-27a-5p and miR-30a-3p (representing the less abundant counterpart of pre-miRNA hairpin known as the passenger strand), overlapped in both profiles, suggesting discordant changes disposed to the acquisition of particular resistance (Supplementary Tables S4 and S5; Supplementary Figures S3 and S4). Genome mapping exposed the preferential polycistronic organization of differentially expressed miRNA genes in HCT-Oxa-c, whereas the miRNA genes in HCT-FU-c were mainly single-transcribed (Supplementary Tables S6 and S7). Indeed, we have found an unusually high 72.5% (29 of 40) proportion of the differential miRNAs representing four clusters in HCT-Oxa-c compared with 17% (2 of 12) in one cluster of HCT-FU-c and 19% of clustered miRNA genes reported in the entire human genome [28]. Our findings raise a possibility that specific cellular factor/s may be dysregulated in the oxaliplatin-resistant subline, which govern transcription of these polycistronic miRNA clusters. According to the estimation of Wang et al. [28], since clustered miRNAs tend to cooperatively control overlapping sets of target genes, these changes could cause the coordinated switch on/off the pathways contributing to the drug resistance.

### 3.3. Comparison of the Selected miRNA Expression in the 2D and 3D Cell Cultures of Drug-Resistant Sublines

The expression patterns of three groups of the most prominent miRNAs prompted by the differential miRNA-Seq were evaluated by quantitative RT-PCR (qRT-PCR) in the four drug-resistant CRC sublines. First, miR-23a-3p, miR-27a-3p, miR-27a-5p and miR-24-2-5p belonging to the intergenic miR-23a/27a/24-2 cluster and miR-23b-3p, miR-27b-3p and miR-27b-5p belonging to the intronic miR-23b/27b/24-1 cluster were selected for further assessment of their potential role in the modulation of oxaliplatin-resistance. Notably, miR-24-3p as well as miR-181b-5p is independently transcribed from two paralogue gene clusters (Supplementary Table S6).

Second, clustered miR-224-5p and miR-452-5p associated with 5-FU resistance resided in the intron of the *GABRE* gene, whereas miR-203b-3p was transcribed independently from its own promoter. Interestingly, only miR-195-5p but not the member of the same miR-497/195 cluster, miR-497-5p, was statistically significant down-regulated in HCT-FU-c (Supplementary Table S7).

Third, as an example of a cluster without high overall variance between sequenced sublines, we verified the three mature constituents, miR-17-5p, miR-19b-3p as well as miR-20a-5p, of polycistronic miR-17/18a/19a/20a/19b-1/92a-1 (also named miR-17-92). This cluster, which is among the most extensively investigated, has been shown to be important for the development of cancer and enhancement of chemo-resistance to cisplatin in human prostate cancer cells [29] or gemcitabine in pancreatic cancer stem cells [30].

As shown in Table 1, the expression of all verified miRNAs presented in the miR-23b/27b/24-1 cluster as well as miR-181-5p were preferentially enhanced in both sublines with acquired resistance to oxaliplatin, notwithstanding that the continuous or pulse drug exposure regimen was used for selection. Polycistronic paralogues miR-23a/27a/24-2, which differ in non-seed target recognition regions, were down-regulated. In contrast, we observed no alterations or even the opposite expression regulation of these miRNAs among the 5-fluorouracil-resistant sublines compared with the parental lines.

qRT-PCR analysis of 5-FU resistance-associated RNAs has found a relevant reduction of miR-224-5p and miR-452-5p in 5-FU- but not in the Oxa-resistant lines. However, miR-203b-3p and miR-195-5p were down regulated only in HCT-FU-c, suggesting the peculiar dysregulations of miRNA expression in the discrete 5-FU-resistant subline. Finally, the representatives of the miR-17–92 cluster, as expected, exposed minor changes and a promiscuous expression pattern across sublines, thus indicating that these miRNAs are unlikely to be the regulators of the drug resistance. Overall, our data verify that miR-23b/27b/24-1, miR-23a/27a/24-2 and miR-181b-5p from polycistronic clusters and miR-224/452 can play a potential role in the acquisition of resistance to oxaliplatin and 5-fluorouracil, respectively. 

The emergence of phenotypic heterogeneity, as a result of spontaneous events or the adaptation of cells to their microenvironment, is frequently observed in genetically homogenous cancer cells [31]. However, as shown in Supplementary Tables S8 and S9, we did not detect significant variations in miRNA expression levels between subclones raised from a single cell, suggesting an unchanging resistance-linked miRNA signature across drug-resistant HCT-Oxa-c or HCT-FU-c cell populations.

To increase the validity of these findings, we examined the miRNA expression profiles in a 3D (spheroid) cell culture system, which might provide a more realistic physiological model of cancerous tissues. Measurement of HCT116 spheroid growth suppression after treatment with various concentrations of the drug revealed the comparable half-maximal growth inhibitory effect at 7.7 µM in 3D models (Supplementary Table S10; Supplementary Figure S5) comparable with IC50 at 4.1 µM in a 2D flat culture for oxaliplatin and 4.4 µM versus 10.5 µM for 5-fluouracil. Growing the HCT-Oxa-c and HCT-FU-c cells in non-adherent conditions has led to spheroid formation with similar morphology to the parental HCT116 (data not shown). Moreover, the transition of HCT-Oxa-c and HCT-FU-c cell lines from a 2D flat culture into 3D spheroids does not substantially change their Oxa and 5-FU resistance (Supplementary Table S10). Notably, the majority of tested miRNAs display no significant differences in expression profiles in 2D and 3D cell culture indicating (Table 1). The discrepancy was only detected for miR-23a-3p and miR-27a-3p due to their slightly reduced amount in the HCT-Oxa-c spheroids.

Taken together, our results highlight the elevated expression of the miR-23b/27b/24-1 cluster and the decreased expression of the miR-224/452 cluster as hallmarks of the lower susceptibility of CRC to Oxa and 5-FU treatment, respectively.

### 3.4. Overexpression of miR-224-5p Promotes the 5-FU-Induced Apoptosis of CRC Cells

The primary miRNA processed into miR-224-5p, miR-224-3p, miR-452-5p and miR-452-3p is encoded within intron 6 of the *GABRE* gene. This gene produces an epsilon subunit of the gamma-aminobutyric acid type A receptor (Supplementary Figure S6A). Our results clearly showed that miR-224/452 cluster expression directly correlates with the alterations in the expression of its host *GABRE* gene mRNA in both HCT-FU-c and DLD-FU-p 5-fluoruracyl-resistant cell lines (Supplementary Table S11).

**Figure 2:**
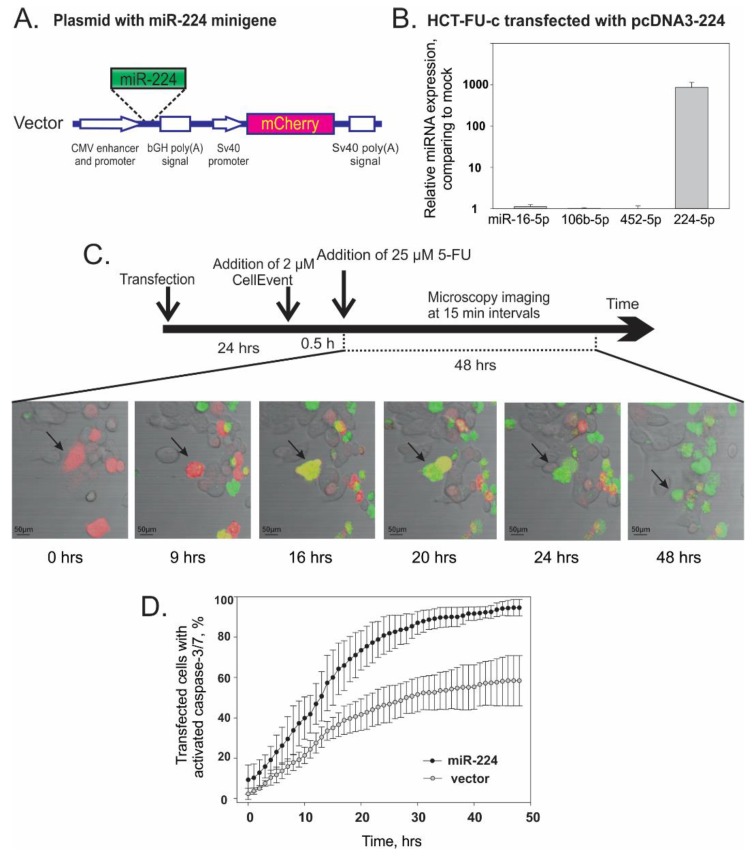
Overexpression of miR-224-5p promotes the apoptosis of HCT-FU-c cells after treatment by 5-fluorouracil. (**A**). A schematic presentation of the cassette for the expression of miR-224 minigene and red fluorescence protein mCherry inserted within the pcDNA3 vector. The miR-224 coding minigene and the reporter gene are placed downstream of CMV and the SV40 promoter, respectively. (**B**). miR-16-5p, miR-106b-5p, miR-452-5p and miR-224-5p relative expression after transfection of HCT-FU-c cells with pcDNA3-224 plasmid comparing with HCT-FU-c cell transfected with vector. RNA was isolated after 24 h. (**C**). Scheme of caspase 3 and 7 activity analysis experiment and representative microscopy images. Cells were transfected 24 h prior to addition of CellEvent^TM^ dye. Then, 30 min after transfected cells were treated with 25 µM 5-FU, the fluorescence of cells was imaged at 15 min intervals for 48 h. Representative microscopy images of the sample captured at different time points. Red signal indicates transfected cells expressing mCherry; green—cells with activated caspase 3/7; yellow—transfected cells with activated caspase 3/7. (**D**). Real-time kinetics of caspase 3/7 activation in cells transfected with miR-224 overexpression plasmid (black dots) or vector (grey dots) in response to 5-FU treatment. The sum effect of the activated caspases is presented as the average ± SD of three biological experiments.

To examine whether the miR-224-5p expression patterns directly impact 5-FU resistance, we have transiently expressed miR-224 as well as reporter mCherry fluorescent protein in the HCT-FU-c cells (Figure 2A). We confirmed that the miR-224-5p level was indeed increased but did not affect control miR-16-5p or miR-106b-5p as well as miR-452-5p, which is transcriptionally linked to miR-224 in the human genome (Figure 2B). The signal from the adjacent mCherry red fluorescence reporter gene constitutively transcribed from SV40 promoter (depicted as red colored cells in Figure 2C) has facilitated the screening of only the cells harboring the plasmid and overexpressing miR-224-5p, thus eliminating data variations caused by different transfection efficiency (25%–40%). In order to track 5-fluorouracil-induced apoptosis, cells were treated with CellEvent^TM^ caspase-3/7 green detection reagent and then caspase 3/7 activities were determined by live-cell time-lapse fluorescence microscopy capturing images at 15-min intervals up to 48 h (green colored cells in Figure 2C). We have identified the precise caspase activation moment for each transfected cell (yellow colored cells in Figure 2C) and performed a time course analysis adding the number of newly caspase-activated cells detected in a certain image to the summary value of apoptotic cells calculated at earlier time points.

As shown in Figure 2D, an obvious difference in caspase-3/7 activation between miR-224-5p up-regulated and the mock transfected cells appeared after 10 h and increased during the remaining time. During 48 h of monitoring, 5-FU-iduced apoptosis were indicated in93% of cells expressing miR-224-5p compared with 58% of apoptotic cells harboring the mCherry reporter vector without observable miRNA. These results suggest that miR-224-5p overexpression increased caspase-3 and -7 activation, and therefore led to the augmentation of 5-FU-dependent cytotoxicity in the HCT-FU-c cells.

### 3.5. CRISPR/Cas9-Mediated Generation and Characterization of miR-23b or miR-27b Knockout Sublines in HCT-Oxa-c Cells

**Figure 3:**
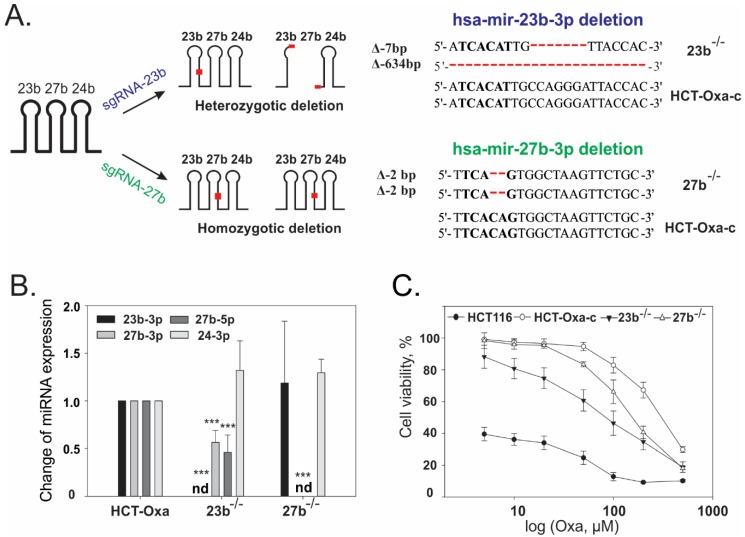
CRISPR/Cas9 mediated knockout of miR-23b or miR-27b gene sensitized HCT-Oxa-c cells to Oxa. (**A**). Mutations in the hsa-miR-23b and hsa-miR-27b genes were identified in both alleles. 7 nt and large 634 nt deletion were determined in miR-23b gene. The 2bp indel was detected in a seed miR-27b-3p sequence. Seed sequences are marked in bold, deletions in red. (**B**) Mutations significantly inhibit the expression of miR-23b-3p, miR-27b-3p and miR-27b-5p. Data normalized compared to HCT-Oxa-c. Relative expression is presented as the average ± SD of three biological experiments. ***, *p* ≤ 0.001. (**C**) Mutations in the miR-23b gene leads to considerable increased HCT-Oxa-c sensitivity to Oxa. miR-27b depletion caused a slight impact to cell viability. Cell viability was determined with the MTT test.

RNA-Seq data revealed a significant 5–10-fold up-regulation of the miR-23b/27b/24-1 cluster in both generated oxaliplatin-resistant sublines. In order to explore its role in chemoresistance, we induced mutations in miR-23b (coding the miR-23b-3p and miR-23b-5p) or miR-27b (miR-27b-3p and miR-27b-5p) genes using the CRISPR/Cas9 technique in the HCT-Oxa cells. In the miR-23b knockout subline, named 23b^−/−^, 7 bp truncation inside of miR-23b-3p and large 634 bp deletion were identified (Figure 3A). The latter covered both miR-23b and miR-27b genes but did not extend to miR-24 (Supplementary Figure S8). The gained subclones completely lacked the expression of miR-23b-3p and also show a 1.8- and 2.2-fold impaired expression of miR-27b-3p and miR-27b-5p, respectively (Figure 3B). In contrast, 2 bp homozygotic microdeletions in a seed miR-27b-3p sequence of 27b^−/−^ subline disrupted only miR-27-3p and miR-27-5p biogenesis without interfering with miR-23b-3p expression. In CRISPR/Cas9 23b^−/−^ and 27b^−/−^ mutants, the level of miR-24-3p expression remained unchanged, indicating that mutations in miR-23b or miR-27b do not affect miR-23b/27b/24-1 cluster biogenesis and disturb only pri-miRNA processing, caused by inefficient Drosha or Dicer cleavage. In addition, we confirmed that the expression of miR-23a-3p and miR-27a-3p, belonging to the paralogical miR-23a/27a/24-2 cluster, remained at the same levels in 23b^−/−^ and 27b^−/−^ mutants compared to parental cells (Supplementary Table S12).

Cluster miR-23b/27b/24-1 is located in the *AP-O* gene intron (Supplementary Figure S6B). Nevertheless, transcription of the host *AP-O* gene does not correlate with the expression level of the miR-23b/27b/24-1 cluster, indicating that indels do not disturb the expression of the *AP-O* gene (Supplementary Table S13).

Finally, we estimated the effect of the loss of the miR-23b and miR-27b expressions in the CRISPR/Cas9 mutants. We found that miR-23b knockout caused a 3.5-fold increase in sensitivity to the Oxa of the HCT-Oxa-c cells, whereas, mutation of miR-27b reversed the resistance considerably less, by 2-fold (Figure 3C; Supplementary Table S14). In contrast, the mutations had little influence on the 5-FU resistance; the IC50 values dropped 1.6- and 1.3-fold in the 23b^−/−^ and 27b^−/−^ cells compared to the HCT-Oxa-c, respectively (Supplementary Table S14).

### 3.6. Identification of miR-23b Targets and Their Functional Bioinformatics Analysis

Since a single miRNA may cause expression changes in hundreds of proteins, and vice versa, a number of miRNAs can regulate the expression of one protein. It is essential to understand which of these changes are relevant to the acquisition of the resistance. In addition, in some cases, miRNAs can impair gene translation without changing RNA abundance, but rather directly attenuating translation machinery [32] and, therefore, the mRNA level rarely correlates with the protein amount. This consideration has prompted us to examine changes in proteomes to identify the genuine targets of miR-23b and to explore their functional and signaling connections.

**Figure 4:**
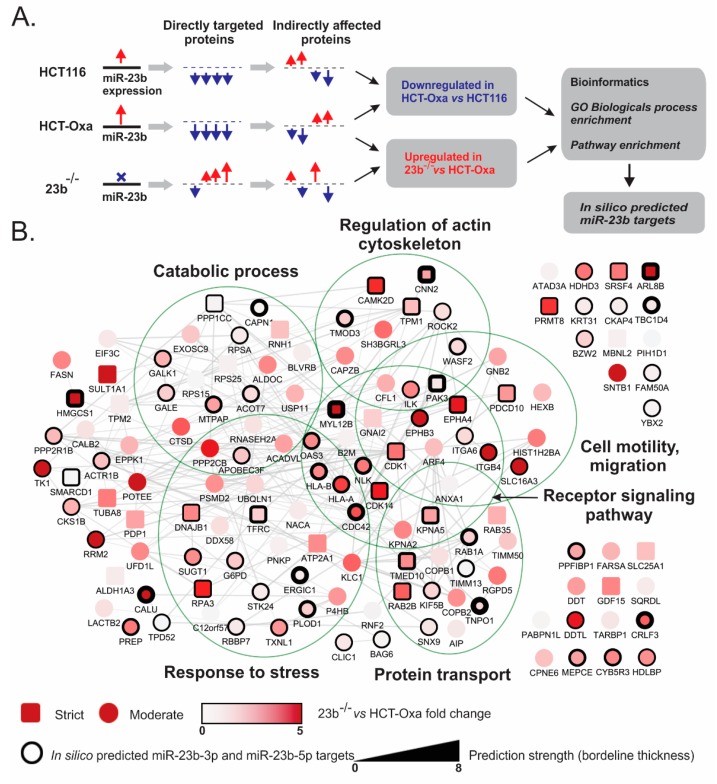
Functional analysis of global proteome changes between HCT116, HCT-Oxa-c and 23b^−/−^ cells. HCT-P, HCT-Oxa-c and 23b^−/−^ cells were subjected to in-depth quantitative proteomic analysis. A protein differential expression, interaction and functional annotation network was built using Cytoscape software. (**A**) The predicted effect of miR-23b on gene expression in various cells and the approach used to analyze omics data. (**B**) Protein networks and enriched functional groups from Gene Ontology (GO) Biological process enrichment analysis. The shape of the nodes represents the strength of target selection conditions, the color demonstrates actual protein-level change in proteomics datasets and the borderline shows mRNA–miRNA interaction stringency, predicted in silico.

To identify direct primary targets and consequential secondary (i.e., indirect) targets of miR-23b, global high-throughput differential quantitative proteomic analysis of HCT116, HCT-Oxa-c and 23b^−/−^ cells has been performed using label-free high-definition mass spectrometry technology. The rationale of target identification and the bioinformatics workflow is outlined in Figure 4A. To define the gene expression directly affected by the miR-23b-3p or mir-23b-5p, multiple criteria have been set: (i) the gene expression has to be increased in miR-23 knockout cells versus Oxa-resistant cells; (ii) the same gene should be down-regulated in Oxa-resistant versus parental HCT116 cells, which express less miR-23b. If a gene met both conditions, we assigned it to the strict targets group. Alternately, if a gene met just the first criteria, we added it to the moderate stringency targets group. In addition, gene consignments to the miR-23b target were strengthened if they were identified in silico using our computation approaches. To find out whether our computation approaches predicted miRNA targets in silico, we compiled a comprehensive in-house dataset, which consolidated the resources of eight publicly available gene target prediction databases, thereby incorporating the benefits of different target prediction algorithms.

Proteomic analysis led to the identification and quantification of a total of 2750 proteins; 269 of these proteins were differentially expressed in HCT-Oxa-c versus HCT116, and 328 proteins were differentially expressed in 23b^−/−^ versus HCT-Oxa-c (Supplementary file 2).

After applying strict and moderate target-sorting conditions, combined with the in silico miR-23b predicted targets, the pooled gene sets were further subjected to functional bioinformatics analysis. Protein interaction networks and functional annotations by Gene Ontology (GO) Biological process enrichment were built and evaluated in Cytoscape software [33] using the stringApp plugin [34]. 

The data show changes in the protein levels that are involved in catabolic processes, response to stress, receptor signaling pathways, regulation of actin cytoskeleton and cell motility and migration. Interestingly, some strong candidates associated with cell resistance to chemotherapy, such as CAMK2D and SRSF4 have, already been implicated in the resistance to platinum compounds [35,36]. Similarly, PDCD10, a predicted miR-23b target, has been shown to play a dual role in cancer cell drug resistance [37] (Figure 4B).

Next, having differential proteomes of parental, Oxa-resistant and miR-23b knockout HCT-116 cell lines allowed us to perform an in-depth bioinformatics analysis and to search for the potential mechanisms of the Oxa resistance. To this end, we performed GO biological process, GO Molecular function and KEGG pathway enrichment of in-silico predicted miR-23b targets and differentially expressed proteins in the miR-23b knockout cell line using ClueGO and CluePedia Cytoscape plug-ins [38,39].

**Figure 5:**
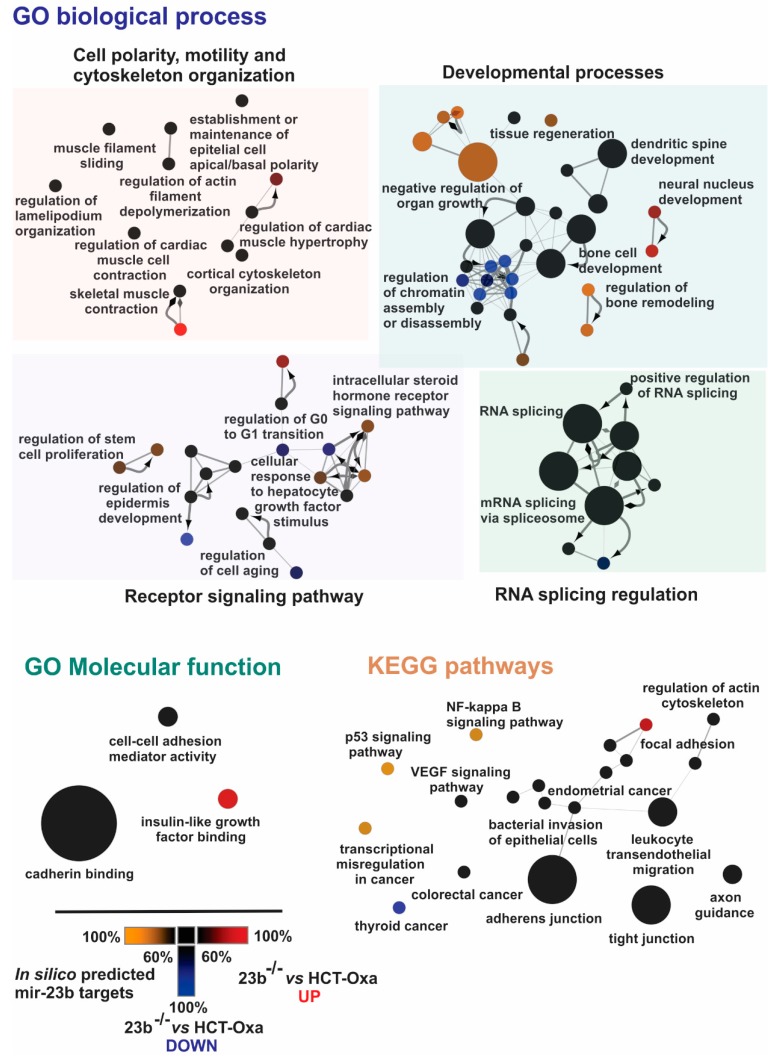
In-depth bioinformatics analysis of differentially expressed proteins and in silico miR-23b targets between Oxa-resistant and miR-23b knockout cell lines. Cell motility, migration, cytoskeleton organization and epithelial–mesenchymal transition-related terms from GO biological process, GO molecular function and KEGG pathway enrichment analysis of in silico-predicted miR-23b targets and differentially expressed proteins in the miR-23b knockout cell line. Terms specifically enriched by in silico-predicted miR-23b targets are designated yellow, terms specifically enriched by 23b^−/−^ vs. HCT-Oxa-c up-regulated proteins in proteomics data are designated red and terms specifically enriched by 23b^−/−^ vs. HCT-Oxa-c down-regulated proteins in proteomics data are designated blue. Terms enriched by all groups are designated black.

The GO biological process enrichment analysis revealed changes in GO terms of cell polarity, motility and cytoskeleton organization as well as changes in the developmental processes, receptor signaling pathways and RNA splicing regulation (Figure 5).

Processes such as cortical cytoskeleton organization (GO:0030865), the regulation of actin filament depolymerization (GO:0030834) and the establishment or maintenance of epithelial cell apical/basal polarity (GO:0045197) are associated with cells undergoing morphological changes and epithelial–mesenchymal transition (EMT) [40,41,42]. These changes suggest that miR-23b is involved in altering the epithelial–mesenchymal state of Oxa-resistant and miR-23b knockout cells. This prediction is also supported by further bioinformatics data. For instance, development-related proteins, cytoskeleton reorganization or EMT as well proteins involved in cell migration are crucial to the biological processes determined in our bioinformatics analysis such as tissue regeneration (GO:0042246), dendritic spine development (GO:0060996) and neural nucleus development (GO:0048857) [43,44,45].

Moreover, we identified changes in receptor signaling pathways resulting in the altered regulation of stem cell proliferation (GO:0072091), epidermis development (GO:0045682), cell aging (GO:0090342) or differences in response to growth factor stimuli (Figure 5). Enrichment analysis highlighted changes in RNA splicing (GO:0008380) and RNA splicing regulation (GO:0043484) and also pointed to EMT as it was previously associated with alterations in RNA splicing mechanisms [46,47]. Furthermore, modified receptor signaling pathways could also result in changes in cell migration and EMT as these processes are regulated by the same receptor tyrosine kinases and other proteins involved in signal transduction [48,49].

GO molecular function enrichment analysis supplemented GO Biological process data by highlighting changes in cell–cell adhesion mediator activity (GO:0098632) and cadherin binding (GO:0045296) (Figure 5). Remarkably, it is well established that the loss of cell–cell junctions and changes in cadherin levels and binding capacity are crucial for EMT [50,51].

Furthermore, as shown in Figure 5, KEGG pathway enrichment also demonstrated similar results such as changes in various signaling pathways including: (i) the NF-kappa B signaling (KEGG:04064) and VEGF signaling pathways (KEGG:04370), (ii) cell–cell and cell–substrate interactions such as in tight junctions (KEGG:04530), adherent junctions (KEGG:04520) or focal adhesion (KEGG:04510) and (iii) the regulation of actin cytoskeleton (KEGG:04810). Notably, all these pathways contribute to the EMT in one way or another [40,41,42,48,52,53].

In summary, the bioinformatics analysis of global differential proteomes shows changes in processes, functions and pathways related to cell motility, migration, cytoskeleton organization, and most importantly related to the epithelial–mesenchymal transition. This allowed us to predict miR-23b involvement in the regulation of complex EMT cellular phenomena contributing to the Oxa resistance mechanism in CRC cells.

### 3.7. miR-23b Contributes to Epithelial Mesenchymal Transition

**Figure 6:**
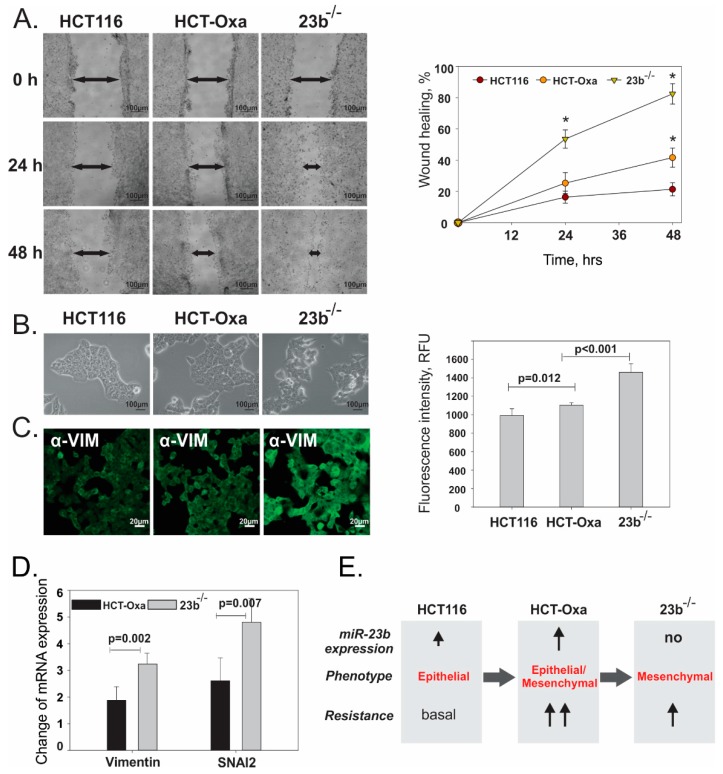
Evaluation of the migration potential and EMT status of HCT116, HCT-Oxa-c and 23b^−/−^ cells. (**A**). Estimation of cell migration capabilities using wound healing assay. (**B**). Identification of cell morphology by bright field microscopy. (**C**). Assessment of mesenchymal marker vimentin by confocal microscopy. (**D**). Analysis of mesenchymal cell marker vimentin and EMT transcription factor *SNAI2* by qPCR. (**E**). Schematic representation of EMT status and resistance of HCT116, HCT-Oxa-c and 23b^−/−^ cells, showing the importance of partial EMT for cancer cell resistance.

Next, we validated proteomic and bioinformatics data that pointed toward the involvement of miR-23b in the regulation of cell EMT as well as their migration. A wound healing assay revealed that 23b^−/−^ cells possess considerably higher migration potential than HCT-Oxa-c cells and HCT116, which exhibit the slowest degree of mobility (Figure 6A). We observed that 23b^−/−^ cells have less cell–cell contact, tend to attach to the surface like fibroblasts and have a higher number of lamellipodia than HCT-Oxa cells (Figure 6B).

Since the increase in cell migration is frequently attributed to the mesenchymal phenotype [41] and our proteomic data also suggest alterations in the EMT of Oxa-resistant cells, we have quantitated a few mesenchymal cell markers to relate their phenotype to miR-23b expression. Analysis of microscopy data supported the results of the wound healing assay, showing the greatest amount of vimentin in 23b^−/−^ cells and a moderately increased amount in HCT-Oxa-c cells compared with HCT116 cells (Figure 6C).

The EMT marker vimentin [54] expression level determined by confocal microscopy and qRT-PCR, as well as the level of one of the EMT-inducing transcription factors *SNAI2* (also known as *SNAIL2* or *SLUG*) [55] determined by qRT-PCR, showed a similar biphasic effect (Figure 6D). Therefore, the acquisition of Oxa resistance coincides with the increase in miR-23b expression, the moderate increase in EMT markers, vimentin and *SNAI2,* expression, with the following rise in their expression in the 23b^−/−^ deficient cells, which are also less resistant to the Oxa treatment.

In summary, these findings show that HCT-Oxa-c cells acquired a hybrid epithelial–mesenchymal phenotype while obtaining resistance to oxaliplatin. Depleting miR-23b from the resistant cell line resulted in decreased drug resistance and a more profound mesenchymal phenotype. This indicates that elevated miR-23b in the Oxa-resistant cells holds them in a hybrid EMT stage and contributes to the cancer cell insensibility to Oxa (Figure 6E).

## 4. Discussion

Chemoresistance is the major obstacle of 5-fluorouracil- and oxaliplatin-based FOLFOX therapy, which is among the most commonly used chemotherapy regiments to treat colorectal and other cancers. Although miRNAs arise as key players responsible for the development of 5-FU and Oxa resistance, most of the miRNA-mediated drug resistance molecular mechanisms remain elusive. To better understand the miRNA-triggered functional processes that contribute to cell adaptation to the Oxa or 5-FU treatment, we have examined the chemoresistance of colorectal cancer cells using different approaches such as miRNAs (2D cultures and 3D spheroids) as well as proteomic profiling and functional analysis of cells overexpressing or knockout of particular miRNA (Figure 1).

Herein, we revealed, for the first time to our knowledge, the miRNA profile of 5-FU or Oxa-resistant HCT116 cancer cells using high-throughput sequencing. We determined 14 differentially expressed miRNAs in HCT-FU-c cells compared to isogenic parental cells, including miR-218-5p, miR-27a-5p, miR-224-5p, miR-195-5p, miR-30a-5p and miR-34a-5p that were previously associated with chemoresistance to 5-FU. It was previously shown that the overexpression of miR-218-5p increased 5-FU cytotoxicity by inhibiting thymidylate synthase and apoptosis suppressor BIRC5 [56]. miR-27a-3p led to sensitization of colorectal cancer cells to the drug by diminishing the expression of dihydropyrimidine dehydrogenase (DPYD), responsible for converting administered 5-FU to the inactive metabolite [57]. Recently, a decrease in miR-195-5p expression has been related to resistance not only to 5-FU, but also to oxaliplatin in HCT116 as well as in RKO cells. Further, proteomic analysis highlighted specific proteins involved in cell division, DNA damage response and nuclear factor kappa-B signaling, which is presumably responsible for chemoresistance [58].

It was reported earlier that miR-224-5p knockout sensitized cells to 5-FU cytotoxicity. Conversely, we registered the down-regulation of this miRNA in 5-FU-resistant lines as well as in all the HCT-FU-c subclones raised from one cell (Table 1; Supplementary Table S9). An exclusively high, 57-fold reduction of the expression was shown in DLD-FU-p cells. Moreover, we observed the significant down-regulation of the miR-224/452 cluster in a 3D spheroids model of HCT-FU-c cells, which more closely recapitulates a tumor’s microenvironment than a 2D monolayer culture [59]. These findings certainly indicated that decreased miR-224-5p expression could be associated with the development of resistance to 5-FU. In accordance with this assumption, we have shown that the overexpression of miR-224-5p in HCT-FU-c significantly increased the number of cells undergoing caspase-3/7-mediated apoptosis after the 5-FU treatment.

At first glance, the present findings contradict the conclusions by Amankwatia et al. [60]. However, in our experiments, both parental lines, HCT116 and DLD-1, harbored a heterozygous point substitution Gly13Asp in the KRAS proto-oncogene, which is mutated in up to 50% cases of CRC [61], while Amankwatia et al. explored the miR-224-5p effect in a HCT116 wild-type KRAS background. The *wt* KRAS line reduction of miR-224-5p expression increased KRAS and BRAF activity, which in turn modulated ERK and AKT phosphorylation and induced chemosensitivity to 5-FU (Supplementary Figure S9). Since the KRAS signaling pathway is already dominant-activated due to the Gly13Asp mutation in HCT116 and DLD1 cells, the miR-224-5p may trigger other factors/pathways, causing enhanced 5-FU resistance in our experiments. In the future, it would be of particular interest to examine the impact of the diminished expression of miR-224-5p on the level of cyclin-dependent kinase inhibitor p21, a key cell cycle regulator in drug-resistant cells. Cell cycle delay for prolonged DNA reparation and synthesis time is among the chemoresistance pathways to protect cancer cells from 5-FU-induced apoptosis [8]. Previously, it was demonstrated that miR-224 negatively regulates p21 expression in colorectal carcinoma [62]. The p21-mediated inhibition of retinoblastoma protein phosphorylation repressed the activity of E2F, leading to the suppression of apoptosis [63]. In addition, a more recent study identified that cytoplasmic p21 phosphorylated at T145 by AKT in complex with Chk2 participates in controlling 5-FU resistance in CRC [64]. Thus, our results suggest that depending on the genetic background, miR-224-5p can be involved in either gaining resistance (Gly13Asp KRAS) or acquiring sensitivity (*wt* KRAS) to 5-FU treatment.

Remarkably, aberrant miR-224-5p expression could also significantly affect chemoresistance to drugs with a different mechanism of action. Indeed, the reduced expression of miR-224-5p led to insensitivity to methotrexate in colorectal cancer cells [65] as well as to imatinib in leukemia cells [66]. Altogether these observations show the important and diverse functions of miR-224-5p in drug resistance.

Interestingly, the host for the miR-224/452 cluster gene *GABRE* exhibited a deregulation signature concordant with miR-224-5p in both HCT-FU-c and DLD-FU-p cell lines (Supplementary Table S11). This gene expression has been reported to be regulated by DNA methylation and proposed as a prominent epigenetic predictor for prostate cancer diagnosis and prognosis [67]. Moreover, positive relationships between *GABRE* and miR-224 levels were reported in hepatocellular carcinoma [68]. Our findings suggest *GABRE* can also be a potential prognostic biomarker for 5-FU-resistant CRC cells.

Much less is known about the roles of miRNA in the development of resistance to Oxa. Here, we identified 40 differentially expressed miRNAs in HCT-Oxa-c cells compared to parental cells. Interestingly, the majority of them (29 of 40 miRNAs, 72.5%) were distributed in miRNA clusters including miR-23a/27a/24-2, miR-23b/27b/24-1, miR-181a-1/181b-1 (or miR-181a-2/181b-2) and miR-379/656. Most of the miRNAs within the same cluster were co-expressed, either being increased or decreased together, suggesting that a common signal, such as transcription factor(s), DNA methylation or histone modification could simultaneously affect multiple miRNAs. Since miRNAs in the same cluster usually regulate covering sets of target genes with similar functions [28], the dysregulation of miRNA clusters can impact a broad range of proteins with related functions. Although the miR-376/656 cluster remains poorly investigated, usually it is down-regulated in cancer and acts like a tumor suppressor [69]; miR-543 could promote EMT and colorectal cancer migration [70]. The miR-181a-1/181b-1 cluster is associated with the development of colorectal cancer, since high levels of miR-181a and miR-181b predict poor overall survival in colorectal cancer patients [71]. The miR-23a/27a/24-2 cluster is clearly associated with colorectal cancer progression [72,73] and chemoresistance [74,75]. However, we found no significant down-regulation of these miRNAs in HCT-Oxa-c 3D cultures, suggesting that they may have little or no impact on chemoresistance to Oxa in our cells.

The expression of miR-23b-3p, miR-27b-3p and miR-27b-5p from the miR-23b/27b/24-1 cluster increased in both oxaliplatin-resistant cell lines, HCT-Oxa-c and HCT-Oxa-p, generated by continuous or pulse selection. Moreover, the increase in expression was observed in different HCT-Oxa-c subclones and in the 3D spheroids. Consistently, miR-27b up-regulation was previously determined in oxaliplatin-resistant HCT116 and LoVo cells [76]. Further, the high expression of miR-27b in plasma was associated with the shorter progression-free survival of colorectal cancer patients after treatment with 5-FU and Oxa [77]. Although the down-regulation of miR-23b-3p expression was detected in colon cancer tissues [78] and plasma samples [79], miR-23b-3p was not previously associated with resistance to Oxa.

In order to gain more insight into the possible mechanisms regulated by the members of the miR-23b/27b/24-1 cluster, we have mutated the genome locus encoding miR-23b and miR-27b using the CRISPR/Cas9 technique in HCT-Oxa cells. We demonstrated that the knockout of miR-23b and minor lowering of miR-27b expression in the 23b^−/−^ cell line showed a greater impact to chemoresistance to Oxa than the down-regulation of miR-27b, which tends to show only a slight effect on cell viability after treatment with oxaliplatin.

Next, to identify targets of miR-23b (miR-23b-3p and miR-23b-5p), we performed a global high-throughput differential proteomic analysis of HCT116, HCT-Oxa-c and 23b^−/−^ cells. Data have linked the miR-23b expression to various biological processes connected to cytoskeleton remodeling, migration and EMT. It has previously been shown that the ectopic expression of miR-23b in HCT116 cells correlated with the acquisition of more profound epithelial phenotypes, the suppression of migration and the diminution of anchorage-dependent programmed cell death (anoikis) resistance [80]. On the other hand, our data demonstrate that in HCT-Oxa-c cells, the miR-23b level is increased and these cells are more motile, mesenchyme and acquired higher Oxa resistance than their parental HCT116 cells. Surprisingly, this apparent contradiction disappears in Oxa-resistant cells with miR-23b knockout, as these cells become even more mesenchyme and motile. However, the elimination of miR-23b expression render Oxa-resistant cells more sensitive to Oxa treatment; this suggests that some balance between cell epithelial–mesenchyme transitions must be kept for the acquisition and maintenance of oxaliplatin resistance. Since various transcription factors are in the dynamic, reciprocal negative interaction with a number of microRNAs [81] may lead to mesenchyme–endothelial phenotype reversal. It is also feasible to predict the existence of intermediate hybrid epithelial–mesenchymal phenotype-containing cells with acquired plasticity and higher resistance to chemotherapy.

Although the aim of this study was to determine predictive markers for the FOLFOX therapy and more specifically for the cell resistance to oxaliplatin or 5-FU, we did not expand our research to clinical material. In our approach, the parental cell lines and their drug-resistant counterparts pre-selected in vitro were used to study the mechanism of drug resistance and to attribute specific miRNAs to the drug insensitivity, making them promising therapeutic targets in future second line therapy.

For this study, we have chosen cell lines with DNA mismatch repair (MMR) system deficiency, although the majority of colorectal tumors are MMR proficient [82]. On the other hand, cancer cells which have a compromised MMR system are less responsive to chemotherapy [83]. Therefore, we have restricted our study to cells that are initially more resistant to drug treatment.

During the study, we focused mostly on cell resistance to oxaliplatin, which is less elucidated than 5-FU. This led to the discovery of a number of miRNAs that correlated with drug insusceptibility. Further analysis highlighted one miRNA (miR-23b) with the biggest contribution to the resistance. We sought to determine its downstream gene targets by knocking out this mRNA in the HCT-116 cell line and performing differential proteomic analysis. Although these studies led us to emphasize the partial EMT role in oxaliplatin resistance, we did not extend the study to other cell lines due to resources.

## 5. Conclusions

In this study, we revealed the profile of miRNAs differentially expressed in 5-FU or Oxa-resistant HCT116 cell lines using global high-throughput sequencing. We identified that miR-224-5p was involved in 5-FU chemoresistance by promoting apoptosis after treatment of HCT-FU-c cells with 5-FU. On the other hand, miR-23b overexpression correlated with Oxa resistance in 2D and 3D cultures and moderate EMT. Remarkably, Oxa-resistant cells, which had miR-23b knockout, became more sensitive to the Oxa treatment and acquired a more mesenchyme cell-like phenotype. This shows that miR-23b participates in the fine balance of EMT and partially contributes to Oxa resistance in CRC.

## Figures and Tables

**Table 1:** Validation of differentially expressed miRNAs in various drug-resistant cell lines by qRT-PCR.

miRNA	Oxaliplatin-Resistant Sublines			5-Fluorouracil-Resistant Sublines			Spheroids	
	HCT-Oxa-c		HCT-Oxa-p	HCT-FU-c		DLD-FU-p	HCT-Oxa-c	HCT-FU-c
	RNA-Seq	qPCR	qPCR	RNA-Seq	qPCR	qPCR	qPCR	qPCR
**Oxaliplatin-resistance related miRNAs**								
23b-3p	up	**5.2 ± 0.9**	**9.5 ± 2.5**	constant	**1.7 ± 0.1**	**−2.1 ± 0.6**	**7.3 ± 3.8**	1.0 ± 0.3
27b-3p	up	**5.9 ± 1.2**	**6.6 ± 1.4**	constant	−1.0 ± 0.2	−1.1 ± 0.1	**5.6 ± 0.4**	1.2 ± 0.3
27b-5p	up	**9.1 ± 1.0**	**5.3 ± 2.2**	constant	n.d.	n.d.	**6.7 ± 1.3**	n.d.
24-3p	constant	−1.1 ± 0.0	**1.6 ± 0.1**	constant	n.d.	n.d.	**1.5 ± 0.1**	n.d.
23a-3p	down	**−1.9 ± 0.4**	−1.2 ± 0.2	constant	−1.1 ± 0.1	1.4 ± 0.3	1.0 ± 0.3	−1.1 ± 0.1
27a-3p	down	**−1.6 ± 0.3**	**−1.6 ± 0.2**	constant	n.d.	n.d.	−1.3 ± 0.1	n.d.
27a-5p	down	−1.6 ± 0.6	**−1.8 ± 0.2**	down	**−1.7 ± 0.2**	1.1 ± 0.1	**−2.0 ± 0.3**	1.2 ± 0.4
24-2-5p	down	**−1.5 ± 0.1**	**−1.6 ± 0.3**	constant	n.d.	n.d.	**−2.0 ± 0.4**	n.d.
181b-5p	up	**2.5 ± 0.4**	**4.9 ± 0.4**	constant	**−1.6 ± 0.1**	1.3 ± 0.2	**2.5 ± 0.5**	−1.5 ± 0.6
**5-FU-resistance related miRNAs**								
203b-3p	constant	−1.3 ± 0.1	**1.9 ± 0.3**	down	**−4.6 ± 1.0**	**1.8 ± 0.2**	n.d.	**−2.5 ± 1.6**
195-5p	constant	**−1.5 ± 0.1**	1.2 ± 0.2	down	**−2.3 ± 0.3**	−1.3 ± 0.1	n.d.	−1.7 ± 0.8
224-5p	constant	1.2 ± 0.2	1.2 ± 0.3	down	**−2.3 ± 0.7**	**−57 ± 16**	−1.2 ± 0.0	**−1.5 ± 0.1**
452-5p	constant	−1.1 ± 0.1	1.1 ± 0.1	down	**−2.9 ± 0.3**	**−3.8 ± 1.0**	−1.2 ± 0.3	−1.5 ± 0.3
**Unbiased miRNAs**								
17-5p	constant	n.d.	n.d.	constant	**1.6 ± 0.3**	1.3 ± 0.1	n.d.	1.4 ± 0.2
19b-3p	constant	n.d.	n.d.	constant	**1.9 ± 0.5**	**1.5 ± 0.1**	n.d.	1.0 ± 0.4
20a-5p	constant	n.d.	n.d.	constant	**1.6 ± 0.1**	−1.5 ± 0.3	n.d.	1.3 ± 0.1

Statistically significant changes are marked in bold, n = 3. n.d., not determined.

## Supplementary Materials

The following are available online at https://www.mdpi.com/2077-0383/8/12/2115/s1, **Supplementary File 1.** A detailed evaluation of differential miRNA-Seq data of each cell line sample assessed in the present study; **Supplementary File 2.** An evaluation of proteomic data of cell lines assessed in the present study; **Supplementary File 3.** Data of GO Biological process, GO Molecular function and KEGG pathways enrichment analysis. **Supplementary Data**. **Figure S1**. Generation of drug-resistant cell lines. **Figure S2.** Effect on cell viability of HCT116, HCT-Oxa-c and HCT-FU-c cell lines after treatment with chemotherapeutical drugs Oxa (A) or 5-FU (B). **Figure S3.** Heatmap of differentially expressed miRNA in HCT-FU-c cell line. **Figure S4**. Heatmap of differentially expressed miRNA in HCT-Oxa-c cell line. **Figure S5**. Relative growth of HCT116, HCT-Oxa-c and HCT-FU-c spheroids after treatment with chemotherapeutical drugs Oxa (A) or 5-FU (B). **Figure S6**. Schematic representation of miR-224/452 (A) and miR-23b/27b/24-1 (B) in relation to the hosts gene GABRE and AP-O respectively. **Figure S7**. Design of sgRNA for miR-23b and miR-27b. **Figure S8**. Deletions in 23b-/- or 27b-/- subclones of HCT-Oxa-c. **Figure S9**. Decreased expression of miR-224-5p can be involved in either gaining resistance (Gly13Asp KRAS) or acquiring sensitivity (wt KRAS) to 5-FU treatment. **Table S1**. Characterization of drug resistant cell sublines. **Table S2**. Summarized miRNA-Seq data statistics for each library. **Table S3**. Statistically significant dysregulated miRNAs determined by RNA-sequencing. **Table S4**. miRNAs with significantly different expression in HCT-Oxa resistant cells. **Table S5**. Deregulated miRNAs in HCT-FU resistant cells. **Table S6**. Polycistronic miRNA clusters differentially expressed in oxaliplatin-resistant cells HCT-Oxa-c. **Table S7**. Differential expressed miRNA clusters in HCT-FU-c resistant cells. **Table S8**. Validation of differentially expressed miRNA in HCT-Oxa-c resistant cells subclones by qPCR. **Table S9**. Validation of differentially expressed miRNA in HCT-FU-c resistant cells subclones by qRT-PCR. **Table S10**. Effect on projected area of HCT116, HCT-Oxa-c and HCT-FU-c cell line spheroids following Oxa or 5-FU treatment. **Table S11**. Correlation of *GABRE* gene expression to miR-224/miR-452 miRNA cluster quantity in various 5-FU resistant cell lines. **Table S12**. miR-23a-3p and miR-27a-3p expression levels in CRISPR/Cas9 mutant’s cells. **Table S13.** *AP-O* gene expression in CRISPR/Cas 9 mutants comparing to HCT116 cell line. **Table S14**. IC50 values of 23b-/- and 27b-/- CRISPR/Cas9 mutants after Oxa or 5-FU treatment. **Table S15**. The list of primer.
