# Supplementary material for: Molecular Insights into miRNA-Driven Resistance to 5-Fluorouracil and Oxaliplatin Chemotherapy: miR-23b Modulates the Epithelial–Mesenchymal Transition of Colorectal Cancer Cells
Source: J Clin Med. 2019 Dec 2;8(12):2115. doi: 10.3390/jcm8122115 (PMC6947029; doi:10.3390/jcm8122115)
Supplement: Supplementary file 1 [file jcm-08-02115-s001.zip › Supplemetary files/Supplementary data.pdf]

SUPPLEMENTARY MATERIAL

Molecular insights into miRNA-driven resistance to 5-fluorouracil and oxaliplatin chemotherapy: miR-23b modulates the endothelial-mesenchymal transition of colorectal cancer cells

Stasė Gasiulė[1,†], Nadežda Dreižė[2,†], Algirdas Kaupinis[2], Raimundas Ražanskas[1], Laurynas Čiupas[1], Vaidotas Stankevičius[1], Žana Kapustina[3], Arvydas Laurinavičius[4,5], Mindaugas Valius[2]* and Giedrius Vilkaitis[1]*

Supplementary Methods

3D cell culture generation and resistance assessment

3D tumor spheroids were generated by seeding cells in non-adherent, agarose coated round-bottomed 96-well plate (5000 cells/well). Cells were centrifuged at 500 g for 10 minutes and left uninterrupted in 5% CO2 atmosphere at 37°C for spheroid to form. After 4 days, spheroids were treated for 48 hours with increasing drug concentrations.

Cell viability in 3D tumor spheroids was established my measuring spheroid size with SpheroidSizer software [1]. All measurements were performed in triplicates and experiment was repeated at least three times.

Sequencing data analysis

Base calling was performed using CASAVA v1.8 (Illumina) and read sequences were outputted in FASTQ format. Quality control of raw sequences was checked using FastQC v0.11.7. Reads were used when quality retain ≥Q30. Next, raw data of FASTQ files were processed with custom R scripts. Briefly, identical sequences were counted and collapsed, adapter sequences were trimmed and reads shorter than 16 nt and longer than 28 nt were removed. The remaining reads were aligned to human miRNA hairpin sequences from miRBase v22 with Bowtie 1.1.2. Additional alignment cycles with modified parameters were performed to include miRNA sequences with 1, 2 or 3 non-template 3'-terminal nucleotides. In addition, any aligned sequence was accepted as mature miRNA if its 5'-terminal mapped to hairpin no further than 1 nt in either direction from the 5' start position of corresponding mature miRNA. Data normalization and differential miRNA expression analyses were performed with R package DeSeq2. After alignment of the reads to currently known pri-miRNAs hairpin precursors annotated in the miRBase database Release 22 [2], a total of 734, 802 and 772 unique miRNA species have been detected in HCT116, HCT-Oxa-c and HCT-FU-c, respectively (Supplementary Table S2). NGS data are available at GEO database (Accession GSE119481).

miRNA target analysis

miRNA–mRNA interaction scores were generated by compiling data from eight interaction databases, which use different interaction prediction algorithms. Data from databases, which contained interaction probability values, were converted to percentiles resulting in arbitrary values from 0 to 1 (or 0.5 to 1 in case were database contained only set of highly probable interactions). Data from databases without any interaction probability values were assigned equal scores by assessing the ratio of all interactions in that database to the count of interactions in our compound database. In case of database with experimental data, interactions with strong experimental evidence were given score 3, and interactions with weak experimental evidence were given score 1. Thus, the scores of resulting 11,592,145 interactions were generated by summing values from: RepTar [3], MiRTar [4], MiRanda [5], PicTar2 [6], MiRMap [7], miRTarget3 [8], TargetScan7 [9] and experimental database miRTARbase [10].

Computational functional analysis of proteomic data

For quantitative analysis of global proteome, statistically significant increase or decrease in protein level was considered as upregulation or downregulation, respectively. The protein-protein interaction network of differentially expressed proteins was built in Cytoscape software [11] using stringApp plugin [12]. Physical interaction data only was used for network generation; no related genes were added to the network. Gene Ontology (GO) Biological process annotations from stringApp were used for enrichment analysis of global proteome changes between HCT116, HCT-Oxa-c and 23b$^{-/-}$ cells.

In-depth bioinformatic analysis was performed using ClueGO and CluePedia Cytoscape plug-ins [13], [14]. GO Biological process, GO Molecular function and KEGG pathways enrichment examination of miR-23b *in silico* predicted targets and differentially expressed proteins in miR-23b knockout cell line was performed. 601 *in silico* predicted miR-23b target proteins, 141 upregulated and 187 downregulated proteins in 23b$^{-/-}$ *versus* HCT-Oxa-c cell lines were subjected to the extensive bioinformatic analysis. A term was considered enriched if at least 10% of the proteins in this population were predicted *in silico* as miRNA targets or at least 4% of proteins were differentially expressed in our proteomics dataset. A term was considered specific to one of the input lists (that is *in silico* targets, upregulated or downregulated proteins) if more that 50% proteins in that term occur in one of the input lists. Terms were connected into groups according to their Kappa score with 0.4 threshold.

Wound healing assay

Cells were seeded at 90-100 % confluence to 6-well plate and allowed to attach overnight. Confluent cultures were scratched with a 200 µl pipette tip and photographed immediately after scratch, after 24 and 48 hours to measure wound healing. Phase-contrast images were analyzed using the MRI Wound Healing Tool for ImageJ. Migration was quantified as the percent of gap closure compared with gap size at the start.

Supplementary Figures

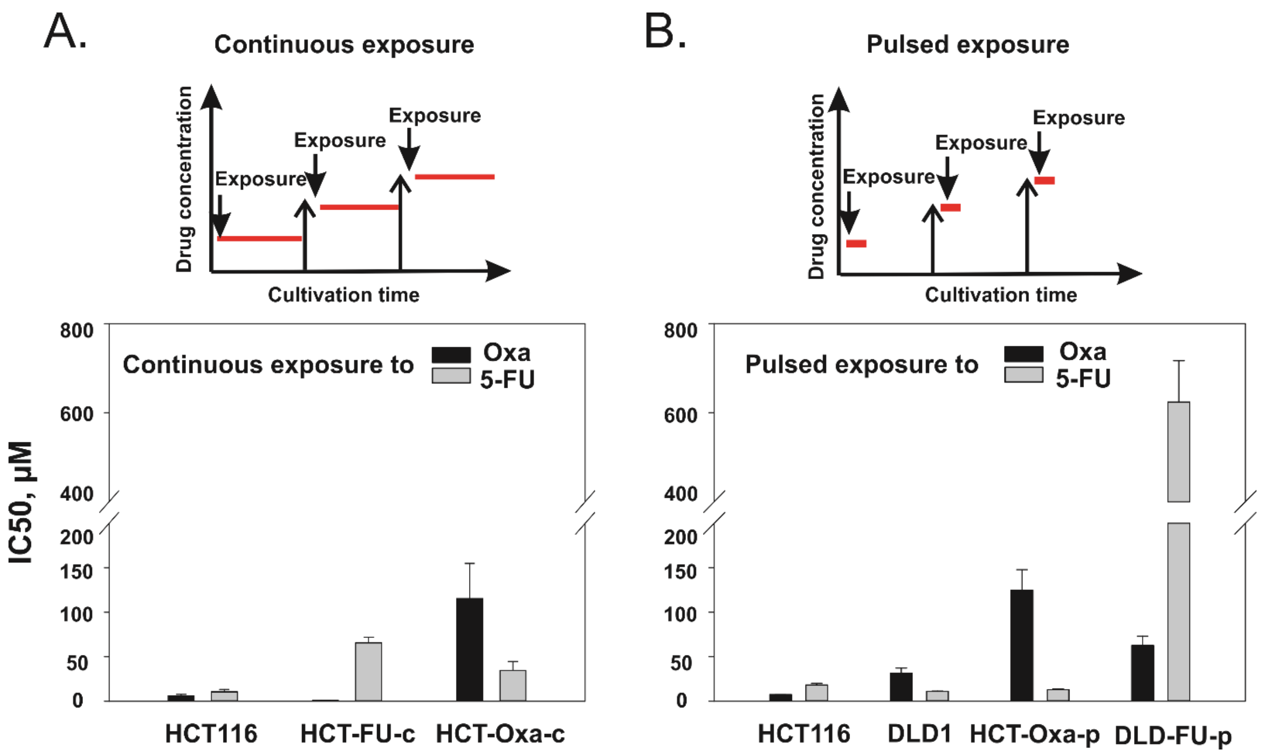

Figure S1. Generation of drug-resistant cell lines. Two different methodological approaches were used for drug-selection of HCT116 or DLD1 cells: continuous (**A**) and pulsed (**B**) exposure to Oxa or 5-FU. IC50 values were determined using MTT assay.

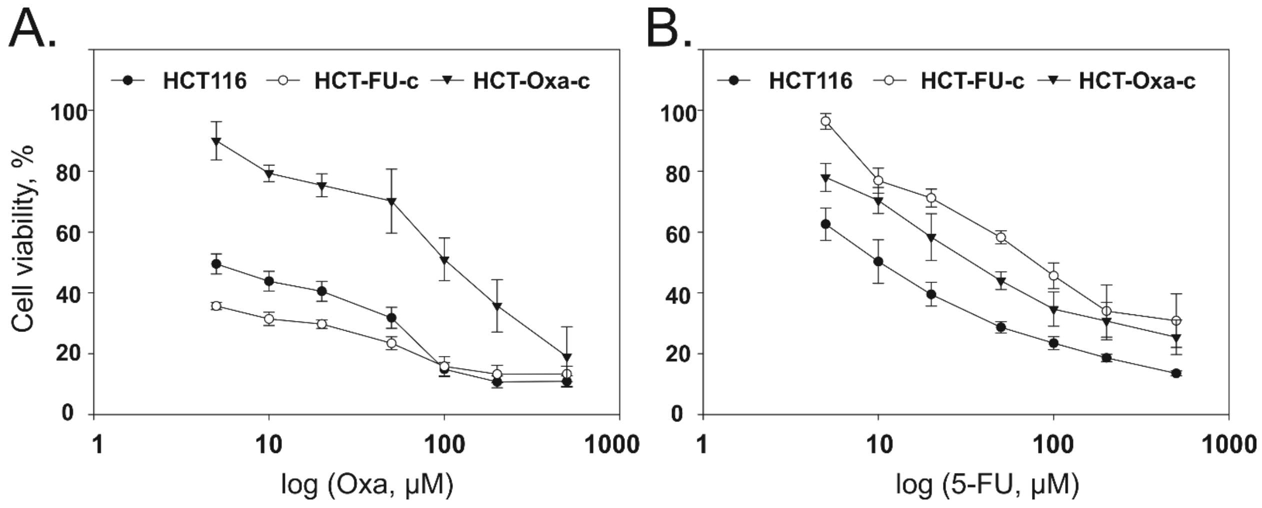

Figure S2. Effect on cell viability of HCT116, HCT-Oxa-c and HCT-FU-c cell lines after treatment with chemotherapeutical drugs Oxa (A) or 5-FU (B). Cell viability were determined by colorimetric MTT assay performed in triplicate.

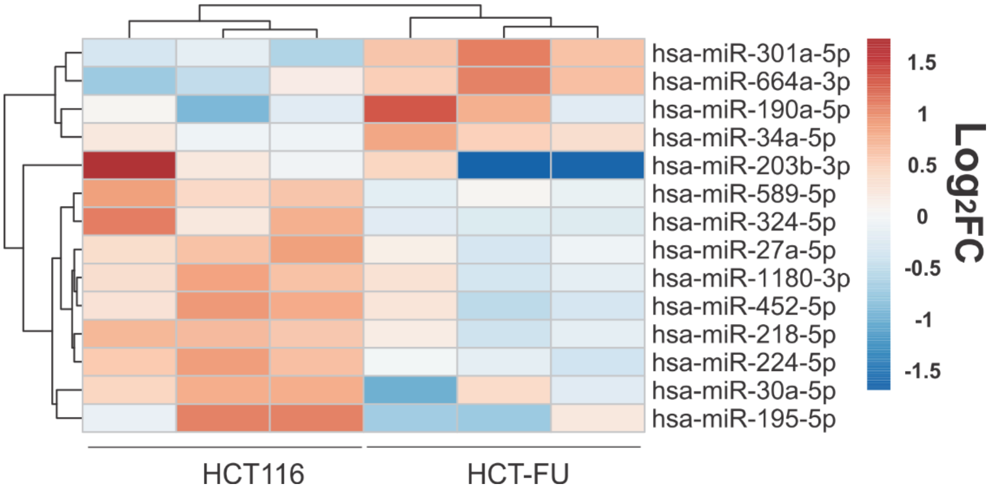

Figure S3. Heatmap of differentially expressed miRNA in HCT-FU-c cell line. Color intensity indicates log-transformed fold change of normalized read counts of corresponding miRNA comparing to normalized read count average from all samples. Fold change, FC > 1.5, $p < 0.05$, RPM > 7.

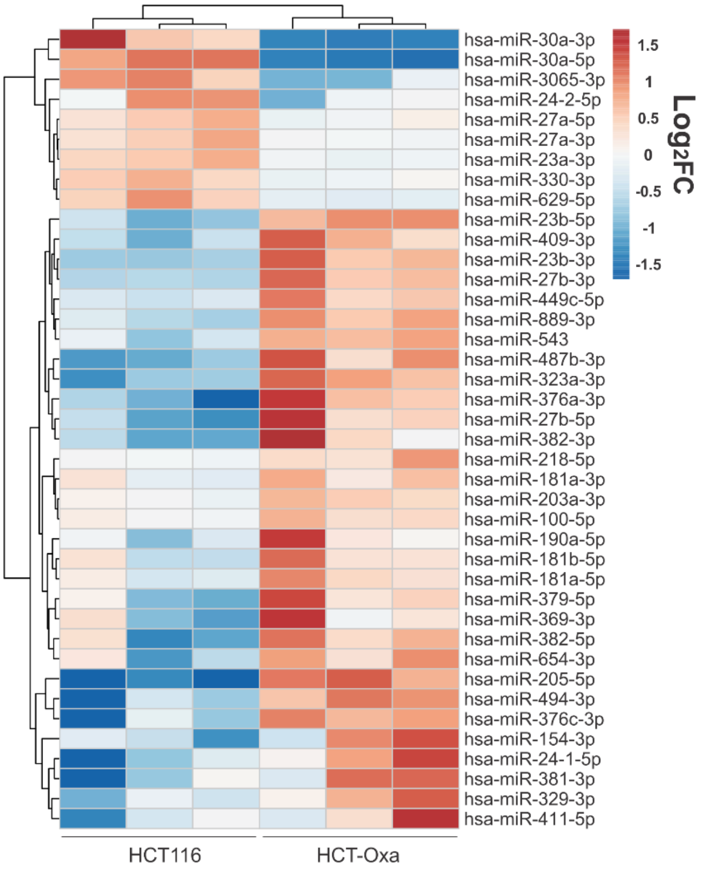

Figure S4. Heatmap of differentially expressed miRNA in HCT-Oxa-c cell line. Color intensity indicates log-transformed fold change of normalized read counts of corresponding miRNA comparing to normalized read count average from all samples. Fold change, FC > 1.5, $p < 0.05$, RPM > 7.

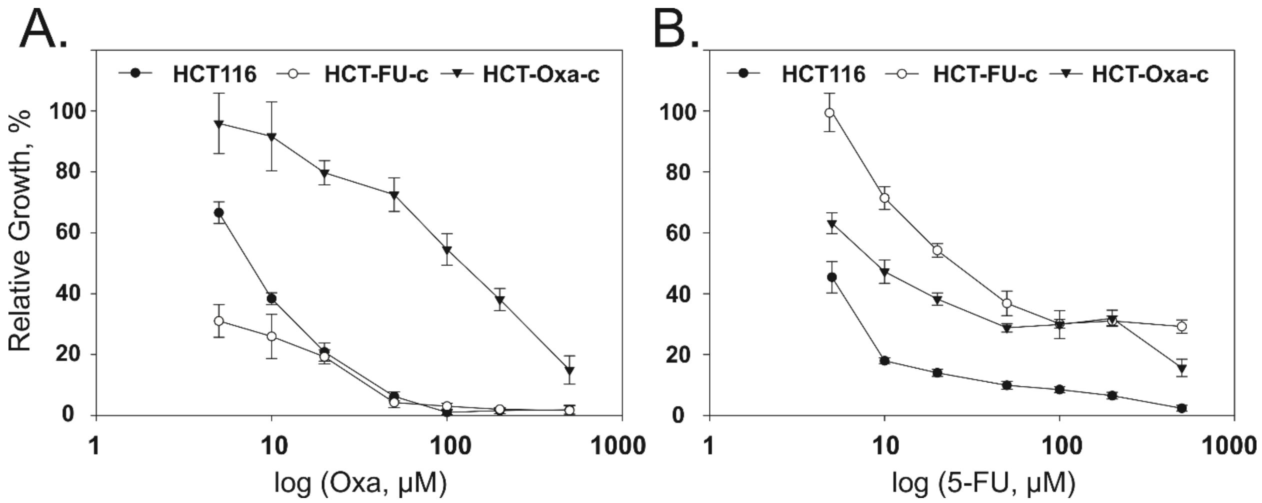

Figure S5. Relative growth of HCT116, HCT-Oxa-c and HCT-FU-c spheroids after treatment with chemotherapeutical drugs Oxa (A) or 5-FU (B). Cells were allowed to form spheroids for 4 days, when treated with Oxa or 5-FU. Relative growth was analyzed by microscopy following 8 days of cell growth, n=3.

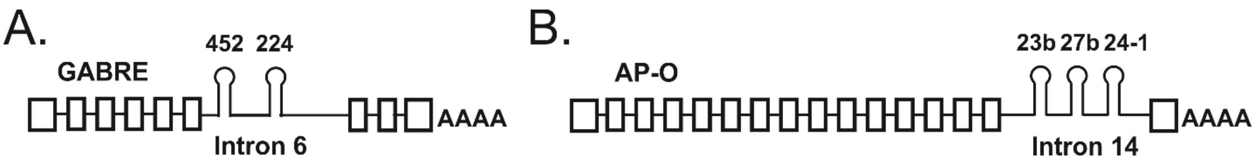

Figure S6. **Schematic representation of miR-224/452 (A) and miR-23b/27b/24-1 (B) in relation to the hosts gene GABRE and AP-O respectively**. miR-224/452 cluster is located in intron 6, miR-23b/27b/24-1- intron 14.

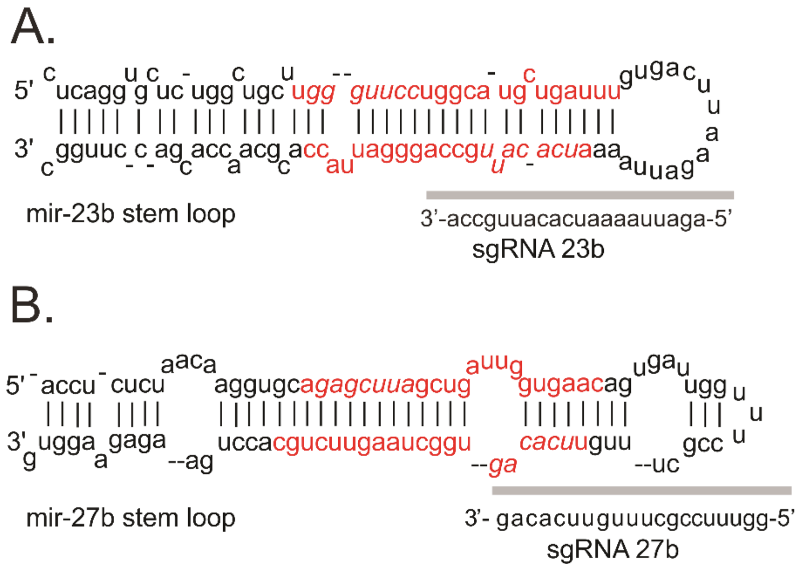

Figure S7. Design of sgRNA for miR-23b and miR-27b. sgRNA were designed by using Benchling program. sgRNA were selected to target guide miRNA strands, miR-23b-3p and miR-27b-3p. Grey line represent sgRNA, red - miRNA, italic – seed sequences.

stemloop of mir-23b
Δ-7bp

5'-CTCAGGTGCTCTGGCTGCTTGGGTTCCTGGCATGCTGATTTGTGACTTAAGATTAAAATCACATTGCC

Δ-634 bp

AGGGATTACCACGCAACCACGACCTTGGCTGCTCCTCCAGAAACCGTGGTCGCGCTCACTGCAGATTGGAG

AACAGGTGCATCTCGTAGCTCTTCTTTGGAAACAAAAGAAGCCACCAGCTGAGGAAGATGCTCACCGGTCACC

stemloop of mir-27b

GTCCCTTTATTTATGCCCAGCGATG**ACCTCTCTAACAAGGTGCAGAGCTTAGCTGATTGGTGAACAGTGAT**

Δ-2bp

TGGTTTCCGCTTTGTTCACAGTGGCTAAGTTCTGCACCTGAAGAGAAGGTGAGATGGGGACAGTTAAGTT

GGAGCCGCTGGGGCAGAGGCCGTTGCTGACGGGCCGGCCGCTGCTGCACAGTCAGCTTGGGTGCGGAGCGCG

ATCCTGGAGGATGAGAGACCACTTGACCCCAAGGATGCACTGTCTCCTGCTGGGAATGCTAGCCATGTACTGA

GTCCTTAAGTCTGTCCACAGAAACATGCACTAATCGGACATCTGTCTGAAAGGTCAAATGTATTGAAAGTTGCA

AAAATTCTTCTTACAAAAAACTAAAACCAAATGCATCACCTAAGTCGTGTGAAATCATGTGGTAGCTCATGGCT

GTGAGCGGGGCGGGGCGGGGCTTTCGGAGGAGCTCCTGTTGTTCTGGGCGCGGTGAACTCTCTCTTGTATTTG

CAGTCCAGGCCTTCGCGTCTCCTGCGCCAGCAGACGGTGCCCACGGAGCTCCCAGCTGAGGCGCTGCTTCTCC

stemloop of mir-24

GGGCTGTCGATTGGACCCGCC**CTCCGGTGCCTACTGAGCTGATATCAGTTCTCATTTTACACACTGGCTCA**

GTTCAGCAGGAACAGGAG-3'

Figure S8. Deletions in 23b$^{-/-}$ or 27b$^{-/-}$ subclones of HCT-Oxa-c. Red boxes highlight 7 bp and 2 bp deletions, while grey box - 634 bp deletion. Stem-loop of miR-23b, miR-27b and miR-24-2 are boldfaced.

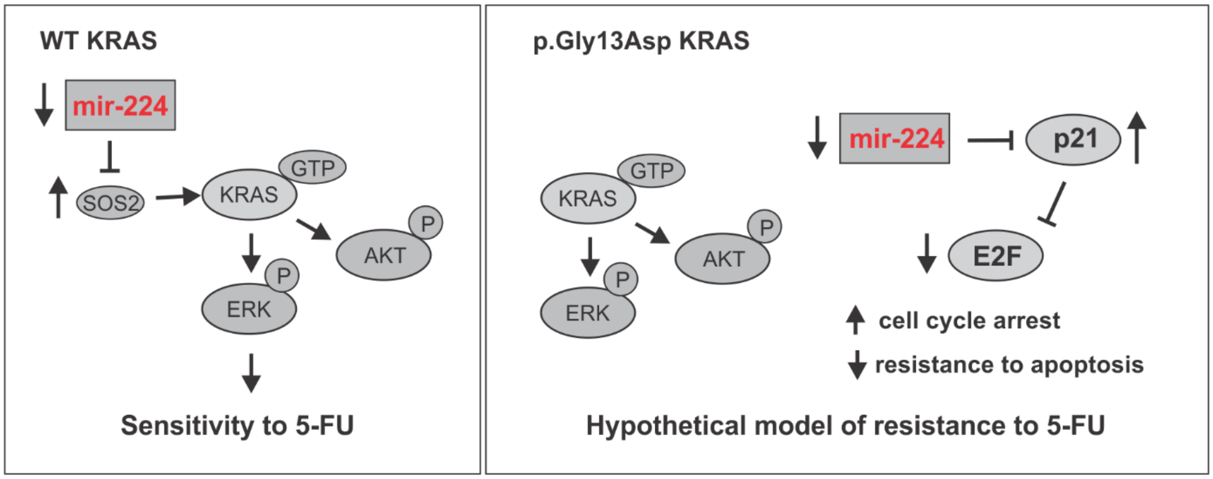

Figure S9. Decreased expression of miR-224-5p can be involved in either gaining resistance (Gly13Asp KRAS) or acquiring sensitivity (*wt* KRAS) to 5-FU treatment. Amankwatia *et al.* [15] reported that in *wt* KRAS line reduction of miR-224-5p expression increased the KRAS and BRAF activity, probably through repression of the Ras/Rho Guanine Nucleotide Exchange Factor gene SOS2, which in turn modulated ERK and AKT phosphorylation and induced chemosensitivity to 5-FU. In Gly13Asp KRAS cell line KRAS signaling pathway is already activated, the miR-224-5p downregulation in HCT-FU-c cells miR-224-5p may trigger other factors such as p21 resulting cell cycle arrest and inhibition of apoptosis through E2F [16-19].

Supplementary Tables

Table S1. Characterization of drug resistant cell sublines. Cell viability were compared using MTT assay. Fold resistance was calculated as IC50 of resistant/IC50 of parental cell line.

**Drug resistant cell subline**	**Selecting drug**	**Exposure regiment**	**Fold resistance**	
			**Oxa**	**5-FU**
Oxa resistant				
HCT-Oxa-c	Oxa	continuous	27.7	3.2
HCT-Oxa-p	Oxa	pulsed	16.2	0.7
5-FU resistant				
HCT-FU-c	5-FU	continuous	0.2	4.5
DLD-FU-p	5-FU	pulsed	1.9	66.2

Table S2. Summarized miRNA-Seq data statistics for each library. Three miRNA libraries of each cell line were sequenced. Reads were aligned to human pri-miRNA hairpins.

**Sample**	**Raw count 16-28 nt**	**Mapped reads to pri-miRNA**	**Mapping rate, %**	**Detected miRNA species**
HCT116_1	1 636 186	1 432 296	88	734
HCT116_2	1 601 846	1 421 828	89	733
HCT116_3	1 098 634	862 726	77	608
HCT-FU_1	1 447 406	1 239 184	86	713
HCT-FU_2	1 700 527	1 496 163	88	802
HCT-FU_3	1 153 810	926 621	80	644
HCT-Oxa_1	2 275 098	1 961 409	86	692
HCT-Oxa_2	2 518 850	2 220 024	88	772
HCT-Oxa_3	1 424 817	1 096 546	77	562

Table S3. Statistically significant dysregulated miRNAs determined by RNA-sequencing. Adjusted fold change > 1.5, p value < 0.05, RPM > 7.

**Resistant cells**	**Dysregulated miRNA**	**Up-regulated**	**Down-regulated**
HCT-Oxa-c	40	31	9
HCT-FU-c	14	4	10

Table S4. miRNAs with significantly different expression in HCT-Oxa resistant cells. Fold change > 1.5, p-value < 0.05, RPM > 7.

No.	miRNA	Base mean, RPM	Fold change	p-value
**Up-regulated**				
1	hsa-miR-205-5p	15	14.37	< 0.001
2	hsa-miR-27b-5p	138	5.68	< 0.001
3	hsa-miR-487b-3p	20	5.64	< 0.001
4	hsa-miR-376a-3p	11	5.04	< 0.001
5	hsa-miR-23b-3p	2244	4.93	< 0.001
6	hsa-miR-323a-3p	13	4.54	< 0.001
7	hsa-miR-382-3p	13	4.22	0.001
8	hsa-miR-27b-3p	8758	4.07	< 0.001
9	hsa-miR-23b-5p	11	3.97	0.001
10	hsa-miR-494-3p	10	3.89	0.002
11	hsa-miR-409-3p	34	3.78	< 0.001
12	hsa-miR-889-3p	34	3.33	< 0.001
13	hsa-miR-376c-3p	8	3.18	0.011
14	hsa-miR-379-5p	58	3.14	0.003
15	hsa-miR-24-1-5p	7	2.97	0.025
16	hsa-miR-154-3p	8	2.71	0.035
17	hsa-miR-381-3p	8	2.69	0.038
18	hsa-miR-543	13	2.69	0.009
19	hsa-miR-329-3p	13	2.62	0.019
20	hsa-miR-382-5p	9	2.60	0.039
21	hsa-miR-411-5p	17	2.54	0.035
22	hsa-miR-369-3p	69	2.52	0.026
23	hsa-miR-449c-5p	20	2.51	0.008
24	hsa-miR-654-3p	11	2.46	0.036
25	hsa-miR-190a-5p	29	2.43	0.027
26	hsa-miR-181b-5p	813	2.09	0.019
27	hsa-miR-181a-5p	1626	1.97	0.010
28	hsa-miR-218-5p	91	1.74	0.034
29	hsa-miR-181a-3p	112	1.70	0.048
30	hsa-miR-203a-3p	3253	1.68	0.009
31	hsa-miR-100-5p	65193	1.54	0.039
**Down-regulated**				
1	hsa-miR-30a-5p	960	-29.84	< 0.001
2	hsa-miR-30a-3p	35	-13.76	< 0.001
3	hsa-miR-3065-3p	9	-2.67	0.037
4	hsa-miR-24-2-5p	98	-2.31	0.016
5	hsa-miR-629-5p	154	-2.20	0.001
6	hsa-miR-23a-3p	9668	-1.90	0.001
7	hsa-miR-330-3p	175	-1.79	0.010
8	hsa-miR-27a-3p	4335	-1.72	0.011
9	hsa-miR-27a-5p	136	-1.66	0.035

Table S5. Deregulated miRNAs in HCT-FU resistant cells. Fold change > 1.5, p-value < 0.05, RPM >7.

No	miRNA	Base mean, RPM	Fold change	p-value
**Up-regulated**				
1	hsa-miR-301a-5p	113	2.42	< 0.001
2	hsa-miR-190a-5p	29	1.84	0.048
3	hsa-miR-664a-3p	12	1.83	0.049
4	hsa-miR-34a-5p	218	1.53	0.049
**Down-regulated**				
1	hsa-miR-224-5p	200	-2.01	0.002
2	hsa-miR-203b-3p	205	-1.91	0.042
3	hsa-miR-324-5p	26	-1.88	0.028
4	hsa-miR-195-5p	15	-1.84	0.049
5	hsa-miR-452-5p	35	-1.80	0.036
6	hsa-miR-218-5p	91	-1.78	0.019
7	hsa-miR-30a-5p	960	-1.77	0.036
8	hsa-miR-27a-5p	136	-1.73	0.017
9	hsa-miR-589-5p	86	-1.73	0.018
10	hsa-miR-1180-3p	155	-1.66	0.036

Table S6. Polycistronic miRNA clusters differentially expressed in oxaliplatin-resistant cells HCT-Oxa-c. **miRNAs in bold** were mis-regulated comparing to parental HCT116. * indicates identical miRNAs localized in a few discrete clusters.

Cluster	Genomic location	pre-miRNA stem-loops	miRNA	Expression
23a/27a/24-2	chr19:13836288-13836651 (-)	3	hsa-miR-23a-5p, **hsa-miR-23a-3p**<br>**hsa-miR-27a-5p, hsa-miR-27a-3p**<br>**hsa-miR-24-2-5p,** hsa-miR-24-3p*	down
23b/27/24-1	chr9:95085226-95086085 (+)	3	**hsa-miR-23b-5p, hsa-miR-23b-3p**<br>**hsa-miR-27b-5p, hsa-miR-27b-3p**<br>**hsa-miR-24-1-5p,** hsa-miR-24-3p*	up
181a-1/181b-1	chr1: 198859044-198859153 (-)	2	**hsa-miR-181a-5p***, **hsa-miR-181a-3p**<br>**hsa-miR-181b-5p***, hsa-miR-181b-3p	up
181a-2/181b-2	chr9: 124692442-124693798 (+)	2	**hsa-miR-181a-5p***<br>hsa-miR-181a-2-3p<br>**hsa-miR-181b-5p***<br>hsa-miR-181b-2-3p	up
379/656	chr14:101022070-101066786 (+)	42	**hsa-miR-379-5p,** hsa-miR-379-3p<br>**hsa-miR-411-5p,** hsa-miR-411-3p<br>hsa-miR-299-5p, hsa-miR-299-3p<br>hsa-miR-380-5p, hsa-miR-380-3p<br>hsa-miR-1197<br>hsa-miR-323a-5p, **hsa-miR-323a-3p**<br>hsa-miR-758-5p, hsa-miR-758-3p<br>329-1:<br>hsa-miR-329-5p*, **hsa-miR-329-3p***<br>329-2:<br>hsa-miR-329-5p*, **hsa-miR-329-3p***<br>hsa-miR-494-5p, **hsa-miR-494-3p**<br>hsa-miR-1193<br>**hsa-miR-543**	up

			hsa-miR-495-5p, hsa-miR-495-3p<br>hsa-miR-376c-5p, **hsa-miR-376c-3p**<br>376a-2:<br>hsa-miR-376a-2-5p, **hsa-miR-376a-3p***<br>hsa-miR-654-5p, **hsa-miR-654-3p**<br>hsa-miR-376b-5p, hsa-miR-376b-3p*<br>376a-1:<br>hsa-miR-376a-5p*, hsa-miR-376a-3p*<br>hsa-miR-300<br>1185-1:<br>hsa-miR-1185-5p*, hsa-miR-1185-1-3p<br>1185-2:<br>hsa-miR-1185-5p*, hsa-miR-1185-2-3p<br>hsa-miR-381-5p, **hsa-miR-381-3p**<br>hsa-miR-487b-5p, **hsa-miR-487b-3p**<br>hsa-miR-539-5p, hsa-miR-539-3p<br>hsa-miR-889-5p, **hsa-miR-889-3p**<br>hsa-miR-544a<br>hsa-miR-655-5p, hsa-miR-655-3p<br>hsa-miR-487a-5p, hsa-miR-487a-3p<br>**hsa-miR-382-5p, hsa-miR-382-3p**<br>hsa-miR-134-5p, hsa-miR-134-3p<br>hsa-miR-668-5p, hsa-miR-668-3p<br>hsa-miR-485-5p, hsa-miR-485-3p<br>hsa-miR-323-5p, hsa-miR-323-3p<br>hsa-miR-154-5p, **hsa-miR-154-3p**<br>hsa-miR-496<br>hsa-miR-377-5p, hsa-miR-377-3p<br>hsa-miR-541-5p, hsa-miR-541-3p<br>hsa-miR-409-5p, **hsa-miR-409-3p**<br>hsa-miR-412-5p, hsa-miR-412-3p<br>hsa-miR-369-5p, **hsa-miR-369-3p**<br>hsa-miR-410-5p, hsa-miR-410-3p<br>hsa-miR-656-5p, hsa-miR-656-3p	
Let-7a-2/100	chr11:122146522<br>-122152296 (-)	2	hsa-let-7a-5p, hsa-let-7a-2-3p<br>hsa-miR-10526-3p<br>**hsa-miR-100-5p**, hsa-miR-100-3p	up
449a/449b/449c	chr5:55170585-<br>55172337 (-)	3	hsa-miR-449a-5p, hsa-miR-449a-3p<br>hsa-miR-449b-5p, hsa-miR-449b-3p<br>**hsa-miR-449c-5p**, hsa-miR-449c-3p	up

Table S7. Differential expressed miRNA clusters in HCT-FU-c resistant cells. miRNAs in bold were mis-regulated comparing to parental HCT116. * indicates identical miRNAs localized in a few discrete clusters.

Cluster	Genomic location	pre-miRNA stem-loops	miRNA	Expres sion
224/452	chrX:151958583-151959699(-)	2	**hsa-miR-224-5p,** hsa-miR-224-3p **hsa-miR-452-5p,** hsa-miR-452-3p	down
195/497	chr17:7017627-7017999-	2	hsa-miR-195-3p**, hsa-miR-195-5p** hsa-miR-497-3p**,** hsa-miR-497-5p	down
23a/27a/24-2	chr19:13836288-13836651 (-)	3	hsa-miR-23a-5p, hsa-miR-23a-3p **hsa-miR-27a-5p**, hsa-miR-27a-3p hsa-miR-24-2-5p, hsa-miR-24-3p*	down

Table S8. Validation of differentially expressed miRNA in HCT-Oxa-c resistant cells subclones by qPCR. Oxa fold resistance was calculated as IC50 of resistant/IC50 of HCT116 cell line. miRNA expression compared to HCT116 cells, n=3. * indicates statistically significant changes.

miRNA	HCT-Oxa-c	Oxa c1	Oxa c2	Oxa c3	Oxa c4
**23a-3p**	-1.9±0.4*	-1.0±0.3	-1.2±0.1	1.0±0.1	-1.3±0.0*
**27a-3p**	-1.6±0.3*	-1.2±0.3	-1.3±0.3	-1.2±0.3	-1.6±0.1*
**23b-3p**	5.2±0.9*	4.2±1.3*	4.4±1.3*	2.8±0.7*	2.1±0.7*
**27b-3p**	5.9±1.2*	4.0±0.5*	4.5±1.1*	2.6±0.4*	2.8±0.8*
**Resistance to Oxa, fold**	14.0	16.0	16.9	9.6	12

Table S9. Validation of differentially expressed miRNA in HCT-FU-c resistant cells subclones by qRT-PCR. 5-FU fold resistance was calculated as IC50 of resistant/IC50 of HCT116 cell line. miRNA expression compared to HCT116 cells, n=3. Based on the unpaired Student's t test, all of these changes were statistically significant.

miRNA	HCT-FU-c	5-FU c1	5-FU c2	5-FU c3
**203b-5p**	-4.6±1.0	-3.9±0.4	-4.0±0.7	-2.0±0.1
**224-5p**	-2.3±0.7	-2.5±0.2	-3.4±0.9	-2.5±0.5
**452-5p**	-2.9±0.3	-2.2±0.2	-1.9±0.2	-2.2±0.2
**Resistance to 5-FU, fold**	3.5	3.9	5.5	23.3

Table S10. Effect on projected area of HCT116, HCT-Oxa-c and HCT-FU-c cell line spheroids following Oxa or 5-FU treatment. Spheroids were analyzed after 8 days treatment with chemotherapeutical drug to perform morphometric analysis (evaluation of shape and size). Oxa and 5-FU concentration causing 50% of size reduction comparing to untreated control were determined.

Cell line	50% size reduction Oxa, μM	Fold change	50% size reduction 5-FU, μM	Fold change
**HCT116**	7.7±0.6		4.4±0.7	
**HCT-Oxa-c**	127±5.6	16.4	10.0±1.7	2.3
**HCT-FU-c**	2.1±0.9	0.3	21.8±1.8	5.0

Table S11. Correlation of *GABRE* gene expression to miR-224/miR-452 miRNA cluster quantity in various 5-FU resistant cell lines. *GUS* housekeeping control were used, n=3. All changes were statistically significant.

Gene	HCT-FU-c	DLD-FU-p
**GABRE**	-2.6±0.2	-84.0±27.6
**miR-224-5p**	-2.3±0.7	-56.5±16.1
**miR-452-5p**	-2.9±0.3	-3.8±1.0

Table S12. miR-23a-3p and miR-27a-3p expression levels in CRISPR/Cas9 mutant's cells. miRNA expression compared to HCT-Oxa-c cells, n=3. *n.d.,* not determined.

Cell line	miR-23a-3p	miR-27a-3p
**23b$^{-/-}$**	1.1±0.3	*n.d*
**27b$^{-/-}$**	1.2±0.4	1.3±0.3

Table S13. *AP-O* gene expression in CRISPR/Cas 9 mutants comparing to HCT116 cell line. *GAPDH* housekeeping control were used, n=3.

Cell line	AP-O
**HCT-Oxa-c**	1.1±0.2
**23b$^{-/-}$**	1.3±0.1
**27b$^{-/-}$**	1.5±0.1

Table S14. **IC50 values of 23b$^{-/-}$ and 27b$^{-/-}$ CRISPR/Cas9 mutants after Oxa or 5-FU treatment**.

Cell line	IC50, Oxa	IC50, 5-FU
**HCT116**	5.2±1.0	6.5±0.8
**HCT-Oxa-c**	313.0±29.4	22.1±3.5
**23b$^{-/-}$**	89.5±22.8	14.2±3.5
**27b$^{-/-}$**	155.0±23.0	17.1±2.9

Table S15. The list of primer.

**RT primers**	
**RNU48**	GTCGTATCCAGTGCAGGGTCCGAGGTATTCGCACTGGATACGACGGCTAGAG
**miR-203b-3p**	GTCGTATCCAGTGCAGGGTCCGAGGTATTCGCACTGGATACGACTCCAGT
**miR-452-5p**	GTCGTATCCAGTGCAGGGTCCGAGGTATTCGCACTGGATACGACTCAGTT
**miR-23b-3p**	GTCGTATCCAGTGCAGGGTCCGAGGTATTCGCACTGGATACGACGTAAT
**miR-23b-5p**	GTCGTATCCAGTGCAGGGTCCGAGGTATTCGCACTGGATACGACAATCC
**miR-27b-3p**	GTCGTATCCAGTGCAGGGTCCGAGGTATTCGCACTGGATACGACCAGAA
**miR-27b-5p**	GTCGTATCCAGTGCAGGGTCCGAGGTATTCGCACTGGATACGACTTCAC
**miR-24-3p**	GTCGTATCCAGTGCAGGGTCCGAGGTATTCGCACTGGATACGACCTGTT
**miR-23a-3p**	GTCGTATCCAGTGCAGGGTCCGAGGTATTCGCACTGGATACGACAAATCC
**miR-27a-3p**	GTCGTATCCAGTGCAGGGTCCGAGGTATTCGCACTGGATACGACGCGGAA
**miR-27a-5p**	GTCGTATCCAGTGCAGGGTCCGAGGTATTCGCACTGGATACGACTGCTCA
**miR-17-5p**	GCCCTCCTTCCAACATAGTACTACCTGC
**miR-19b-3p**	CCCTACCGCGCAATAAACTTCAGTTTTGC
**miR-20a-5p**	TTCATTTGTTAGCGAGCGGCTACCTGCA
**miR-16-5p**	CCTTTGAGGTTGGTACTACGGCGCCAATA
**miR-106b-5p**	GTGCAGTATAGGTACGGCTATCTGCAC
**miR-224-5p**	GTAAGCACGCTACATCCTGATAAACGGAAC
**miR-195-5p**	GTGTAGGCCCAATACCAGAGCCAATATT
**qPCR miRNA primers**	
**miR-224-5-p-FW**	CGTTTGCCAAGTCACTAGTGGT
**miR-224-5-p REV**	TTGTAAGCACGCTACATCCTGA
**miR-195-5p-FW**	GGAGTGTAGGCCCAATACCAGA
**miR-195-5p-REV**	TGCCACTTAGCAGCACAGAAA
**miR-17-5p FW**	ACCCTCCAAAGTGCTTACAGTG
**miR-17-5p REV**	CTTGCCCTCCTTCCAACATAGT
**miR-19b-3p-FW**	AATCCCTACCGCGCAATAAACT
**miR-19b-3p-REV**	CCGCTGTGCAAATCCAGTC
**miR-20a-5p-FW**	CGCCATGTAAAGTGCTTATAGTGC
**miR-20a-5p-REV**	CGATTCATTTGTTAGCGAGCGG
**miR-16-5p FW**	ACCTTTGAGGTTACTACGG
**miR-16-5p REV**	GTGCAGTAGCAGCACGTAAAT
**miR-106b-5p FW**	ATCGTGCAGTATAGGTACGGCT
**miR-106b-5p REV**	CCGCGTAAAGTGCTGACAGT
**miR-27b-3p FW**	CCGGCTAACTAACCACGATTCT
**miR-27b-3p REV**	CTTTGTTTCCTTCACAGTGGCTAA
**miR-24-3p FW**	CGCACATGACTCGTAGATACGG
**miR-24-3p REV**	TGCGTGGCTCAGTTCAGC
**miR-452-5p-FW**	GCGGAACTGTTTGCAGAGG
**miR-23b-3p FW**	GCGATCACATTGCCAGGG
**miR-23b-5p FW**	CTGGGTTCCTGGCATGC
**miR-27b-5p FW**	GGCGAGAGCTTAGCTGATTG
**miR-27a-3p FW**	GGCGTTCACAGTGGCTAAG
**miR-27a-5p-FW**	GCGAGGGCTTAGCTGCTTG
**miR-203b-3p-FW**	CGGCGTTGAACTGTTAAGAACC
**miR-27a-5p-FW**	GCGAGGGCTTAGCTGCTTG
**RNU48-FW**	GCGTGCCATCACCGCAGC
**Universal REV**	GTGCAGGGTCCGAGGT

**qPCR mRNA primers**	
**GUS FW**	GAAAATATGTGGTTGGAGAGCTCATT
**GUS REV**	AGACCAGGCAGGGGAGTAAC
**GABRE FW**	TCCCAGACCTGGTACGACG
**GABRE REV**	TGCTCGTGGGTCCTCTTAGAA
**APO FW**	TCGAGGATGGAAACAGATTCAAG
**APO REV**	TTTGTCACGGGAATATGGCAG
**GAPDH FW**	GGAGCGAGATCCCTCCAAAAT
**GAPDH REV**	GGCTGTTGTCATACTTCTCATGG
**SNAI2 FW**	CGAACTGGACACACATACAGTG
**SNAI2 REV**	CTGAGGATCTCTGGTTGTGGT
**Vimentin FW**	CCTTGAACGCAAAGTGGAATC
**Vimentin REV**	CCTTGAACGCAAAGTGGAATC
**miR-224 cloning**	
**224-FW**	CAGGGGAAGCTTCAGAGGGCTGGGCTAC
**224-REV**	GGAGAGGGATCCACCTGCCCCAGGGCCC
**specific to pcDNA3 vector**	
**T7**	TAATACGACTCACTATAGGG
**BGH**	TAGAAGGCACAGTCGAGG
**SV40-FW**	CCCCATGGCTGACTAATTTT
**SV40-REV**	GTGAAATTTGTGATGCTATTGC
**pMTCdir1**	TGGCACCAAAATCAACGGGAC
**SV40dir**	CCCCATGGCTGACTAATT
**sgRNA sequence**	
**sgRNA-23b**	AGATTAAAATCACATTGCCA
**sgRNA-27b**	GGTTTCCGCTTTGTTCACAG
**sgRNA cloning**	
**sgRNA-23b FW**	CACCGAGATTAAAATCACATTGCCA
**sgRNA-23b REV**	AAACTGGCAATGTGATTTTAATCTC
**sgRNA-27b FW**	CACCGGGTTTCCGCTTTGTTCACAG
**sgRNA-27b REV**	AAACCTGTGAACAAAGCGGAAACCC
**PAGE analysis**	
**23b PAGE FW**	CTGCTCTCAGGTGCTCTG
**23b PAGE REV**	TCCAATCTGCAGTGAGCG
**27b PAGE FW**	TCACCGTCCCTTTATTTATGCC
**27b PAGE REV**	CCAGCGGCTCCAACTTAA
**miR-23b/27b/24-1 cluster amplification**	
**miR-23b/27b/24-1 FW**	CTGCTCTCAGGTGCTCTG
**miR-23b/27b/24-1 REV**	CTCCTGTTCCTGCTGAACTGAG
**pSpCas9(BB)-2A-Puro specific vector**	
**U6 dir**	TAGAAGGCACAGTCGAGG
**pUC19 specific vector**	
**pUC19 dir**	GCCAGGGTTTTCCCAGTCACGA
**pUC19 REV**	GAGCGGATAACAATTTCACACAGG

Supplementary References

1. Chen, W. J., C. Wong, E. Vosburgh, A. J. Levine, D. J. Foran, and E. Y. Xu. "High-Throughput Image Analysis of Tumor Spheroids: A User-Friendly Software Application to Measure the Size of Spheroids Automatically and Accurately." *Jove-Journal of Visualized Experiments*, no. 89 (2014).
2. Kozomara, A., and S. Griffiths-Jones. "Mirbase: Annotating High Confidence Micrornas Using Deep Sequencing Data." *Nucleic Acids Research* 42, no. D1 (2014): D68-D73.
3. Elefant, N., A. Berger, H. Shein, M. Hofree, H. Margalit, and Y. Altuvia. "Reptar: A Database of Predicted Cellular Targets of Host and Viral Mirnas." *Nucleic Acids Research* 39 (2011): D188-D94.
4. Hsu, J. B. K., C. M. Chiu, S. D. Hsu, W. Y. Huang, C. H. Chien, T. Y. Lee, and H. D. Huang. "Mirtar: An Integrated System for Identifying Mirna-Target Interactions in Human." *Bmc Bioinformatics* 12 (2011).
5. Enright, A. J., B. John, U. Gaul, T. Tuschl, C. Sander, and D. S. Marks. "Microrna Targets in Drosophila." *Genome Biology* 5, no. 1 (2004).
6. Krek, A., D. Grun, M. N. Poy, R. Wolf, L. Rosenberg, E. J. Epstein, P. MacMenamin, I. da Piedade, K. C. Gunsalus, M. Stoffel, and N. Rajewsky. "Combinatorial Microrna Target Predictions." *Nature Genetics* 37, no. 5 (2005): 495-500.
7. Vejnar, C. E., and E. M. Zdobnov. "Mirmap: Comprehensive Prediction of Microrna Target Repression Strength." *Nucleic Acids Research* 40, no. 22 (2012): 11673-83.
8. Wang, X. W. "Improving Microrna Target Prediction by Modeling with Unambiguously Identified Microrna-Target Pairs from Clip-Ligation Studies." *Bioinformatics* 32, no. 9 (2016): 1316-22.
9. Agarwal, V., G. W. Bell, J. W. Nam, and D. P. Bartel. "Predicting Effective Microrna Target Sites in Mammalian Mrnas." *Elife* 4 (2015).
10. Chou, C. H., S. Shrestha, C. D. Yang, N. W. Chang, Y. L. Lin, K. W. Liao, W. C. Huang, T. H. Sun, S. J. Tu, W. H. Lee, M. Y. Chiew, C. S. Tai, T. Y. Wei, T. R. Tsai, H. T. Huang, C. Y. Wang, H. Y. Wu, S. Y. Ho, P. R. Chen, C. H. Chuang, P. J. Hsieh, Y. S. Wu, W. L. Chen, M. J. Li, Y. C. Wu, X. Y. Huang, F. L. Ng, W. Buddhakosai, P. C. Huang, K. C. Lan, C. Y. Huang, S. L. Weng, Y. N. Cheng, C. Liang, W. L. Hsu, and H. D. Huang. "Mirtarbase Update 2018: A Resource for Experimentally Validated Microrna-Target Interactions." *Nucleic Acids Research* 46, no. D1 (2018): D296-D302.
11. Shannon, P., A. Markiel, O. Ozier, N. S. Baliga, J. T. Wang, D. Ramage, N. Amin, B. Schwikowski, and T. Ideker. "Cytoscape: A Software Environment for Integrated Models of Biomolecular Interaction Networks." *Genome Research* 13, no. 11 (2003): 2498-504.
12. Szklarczyk, D., J. H. Morris, H. Cook, M. Kuhn, S. Wyder, M. Simonovic, A. Santos, N. T. Doncheva, A. Roth, P. Bork, L. J. Jensen, and C. von Mering. "The String Database in 2017: Quality-Controlled Protein-Protein Association Networks, Made Broadly Accessible." *Nucleic Acids Research* 45, no. D1 (2017): D362-D68.
13. Bindea, G., B. Mlecnik, H. Hackl, P. Charoentong, M. Tosolini, A. Kirilovsky, W. H. Fridman, F. Pages, Z. Trajanoski, and J. Galon. "Cluego: A Cytoscape Plug-in to Decipher Functionally Grouped Gene Ontology and Pathway Annotation Networks." *Bioinformatics* 25, no. 8 (2009): 1091-93.
14. Bindea, G., J. Galon, and B. Mlecnik. "Cluepedia Cytoscape Plugin: Pathway Insights Using Integrated Experimental and in Silico Data." *Bioinformatics* 29, no. 5 (2013): 661-63.
15. Amankwatia, E. B., P. Chakravarty, F. A. Carey, S. Weidlich, R. J. C. Steele, A. J. Munro, C. R. Wolf, and G. Smith. "Microrna-224 Is Associated with Colorectal Cancer Progression and Response to 5-Fluorouracil-Based Chemotherapy by Kras-Dependent and -Independent Mechanisms." *British Journal of Cancer* 112, no. 9 (2015): 1480-90.
16. Karimian, A., Y. Ahmadi, and B. Yousefi. "Multiple Functions of P21 in Cell Cycle, Apoptosis and Transcriptional Regulation after DNA Damage." *DNA Repair* 42 (2016): 63-71.
17. Jung, Y. S., Y. J. Qian, and X. B. Chen. "Examination of the Expanding Pathways for the Regulation of P21 Expression and Activity." *Cellular Signalling* 22, no. 7 (2010): 1003-12.
18. Olaru, A. V., S. Yamanaka, C. Vazquez, Y. Mori, Y. L. Cheng, J. M. Abraham, T. M. Bayless, N. Harpaz, F. M. Selaru, and S. J. Meltzer. "Microrna-224 Negatively Regulates P21 Expression

During Late Neoplastic Progression in Inflammatory Bowel Disease." *Inflammatory Bowel Diseases* 19, no. 3 (2013): 471-80.
19. Maiuthed, A., C. Ninsontia, K. Erlenbach-Wuensch, B. Ndreshkjana, J. K. Muenzner, A. Caliskan, P. H. Ahmed, C. Chaotham, A. Hartmann, A. V. Roehe, V. Mahadevan, P. Chanvorachote, and R. Schneider-Stock. "Cytoplasmic P21 Mediates 5-Fluorouracil Resistance by Inhibiting Pro-Apoptotic Chk2." *Cancers* 10, no. 10 (2018).
